# Pharmacogenomics of cisplatin‐induced neurotoxicities: Hearing loss, tinnitus, and peripheral sensory neuropathy

**DOI:** 10.1002/cam4.4644

**Published:** 2022-03-23

**Authors:** Xindi Zhang, Matthew R. Trendowski, Emma Wilkinson, Mohammad Shahbazi, Paul C. Dinh, Megan M. Shuey, Regeneron Genetics Center, Darren R. Feldman, Robert J. Hamilton, David J. Vaughn, Chunkit Fung, Christian Kollmannsberger, Robert Huddart, Neil E. Martin, Victoria A. Sanchez, Robert D. Frisina, Lawrence H. Einhorn, Nancy J. Cox, Lois B. Travis, M. Eileen Dolan

**Affiliations:** ^1^ Department of Medicine University of Chicago Chicago Illinois USA; ^2^ Division of Medical Oncology Indiana University Indianapolis Indiana USA; ^3^ Department of Medicine Vanderbilt University Medical Center Nashville Tennessee USA; ^4^ Department of Medical Oncology Memorial Sloan‐Kettering Cancer Center New York New York USA; ^5^ Department of Surgical Oncology Princess Margaret Cancer Centre Toronto Ontario Canada; ^6^ Department of Medicine University of Pennsylvania Philadelphia Pennsylvania USA; ^7^ J.P. Wilmot Cancer Institute University of Rochester Medical Center Rochester New York USA; ^8^ Division of Medical Oncology University of British Columbia Vancouver British Columbia Canada; ^9^ Royal Marsden Hospital London UK; ^10^ Department of Radiation Oncology Dana‐Farber Cancer Institute Boston Massachusetts USA; ^11^ Department of Otolaryngology – Head and Neck Surgery University of South Florida Tampa Florida USA; ^12^ Departments of Medical Engineering and Communication Sciences and Disorders, Global Center for Hearing and Speech Research University of South Florida Tampa Florida USA; ^13^ Department of Epidemiology, Fairbanks School of Public Health Indiana University Indianapolis Indiana USA

**Keywords:** cisplatin, GWAS, ototoxicity, survivorship, neurotoxicity, testicular cancer

## Abstract

**Purpose:**

Cisplatin is a critical component of first‐line chemotherapy for several cancers, but causes peripheral sensory neuropathy, hearing loss, and tinnitus. We aimed to identify comorbidities for cisplatin‐induced neurotoxicities among large numbers of similarly treated patients without the confounding effect of cranial radiotherapy.

**Methods:**

Utilizing linear and logistic regression analyses on 1680 well‐characterized cisplatin‐treated testicular cancer survivors, we analyzed associations of hearing loss, tinnitus, and peripheral neuropathy with nongenetic comorbidities. Genome‐wide association studies and gene‐based analyses were performed on each phenotype.

**Results:**

Hearing loss, tinnitus, and peripheral neuropathy, accounting for age and cisplatin dose, were interdependent. Survivors with these neurotoxicities experienced more hypertension and poorer self‐reported health. In addition, hearing loss was positively associated with BMIs at clinical evaluation and nonwork‐related noise exposure (>5 h/week). Tinnitus was positively associated with tobacco use, hypercholesterolemia, and noise exposure. We observed positive associations between peripheral neuropathy and persistent vertigo, tobacco use, and excess alcohol consumption. Hearing loss and *TXNRD1,* which plays a key role in redox regulation, showed borderline significance (*p* = 4.2 × 10^−6^) in gene‐based analysis. rs62283056 in *WFS1* previously found to be significantly associated with hearing loss (*n* = 511), was marginally significant in an independent replication cohort (*p* = 0.06; *n* = 606). Gene‐based analyses identified significant associations between tinnitus and *WNT8A* (*p* = 2.5 × 10^−6^)*,* encoding a signaling protein important in germ cell tumors.

**Conclusions:**

Genetics variants in *TXNRD1 and WNT8A* are notable risk factors for hearing loss and tinnitus, respectively. Future studies should investigate these genes and if replicated, identify their potential impact on preventive strategies.

## Graphical abstract

Cisplatin‐induced toxicities in testicular cancer survivors and their association with clinical characteristics (body mass index, hypertension, and hypercholesterolemia) were evaluated along with the effect of modifiable factors (smoking status, alcohol consumption, and noise exposure) on risk of these toxicities. Genetic analysis identified genes that make certain individuals at risk for hearing loss or tinnitus after treatment with this drug.

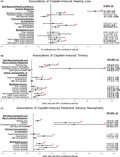

## 1 INTRODUCTION

Cisplatin and other platinating agents represent the most widely used and successful class of cytotoxic drugs worldwide. More than 5.8 million patients (pediatric and adults) globally are diagnosed each year with cancers (e.g., testicular, ovarian, bladder, lung, head and neck, pancreas, breast, endometrium, esophagus, advanced cervical cancer, lymphomas, metastatic osteosarcoma, and others) for which first‐line therapy can potentially include platinating agents.^1^, ^2^ Cisplatin is associated with over 95% 5‐year survival rates in germ cell tumors, but can result in debilitating off target effects including ototoxicity, neurotoxicity, nephrotoxicity, and cardiometabolic abnormalities.^3^, ^4^ Unfortunately, there are no approved preventive measures and no FDA‐approved drug therapies for these toxicities; however, patients would benefit from individualized risk assessments, allowing for detailed education and counseling, and the development of a personalized treatment and monitoring plan. There are attempts to identify individuals a priori who are more likely to develop these sequelae through studies of genetic risk factors and nongenetic comorbidities.^5^, ^6^ However, clinical implementation of assessing genetic biomarkers and counseling patients with nongenetic comorbidities with regard to potential toxicities has been limited.

To ascertain both genetic risk factors and modifiable comorbidities for cisplatin‐induced toxicities, we constructed the Platinum Study, a well‐phenotyped cohort of testicular cancer survivors (TCS) treated with homogenous cisplatin‐based chemotherapy,^7^, ^8^ which consisted of primarily four cycles of EP (etoposide and cisplatin) or three cycles of BEP (bleomycin, etoposide, and cisplatin).^8^ Testicular cancer is the most common malignancy among young men, predominantly of European descent.^9^ Survivors can subsequently live upwards of 50 years following treatment, further accentuating the debilitating effects of iatrogenic neurotoxicities. Cisplatin‐induced neurotoxicities are common, long term, irreversible adverse events in TCS, with 75%–80% of survivors developing hearing loss,^10^, ^11^ 40% experiencing tinnitus,^12^ and 56% reporting symptoms of peripheral sensory neuropathy.^13^

Through an agnostic genome‐wide association study (GWAS), a single nucleotide polymorphism (SNP) in *WFS1* (rs62283056; *p* = 1.4 × 10^−8^) was associated with increased susceptibility to cisplatin‐induced hearing loss,^11^ and replicated in an independent Canadian study of 229 TCS evaluating the same phenotype (*p* = 5.67 × 10^−3^).^14^ Although a traditional GWAS of cisplatin‐induced tinnitus found no genome‐wide significant signals, *OTOS* (rs7606353; *p* = 1.90 × 10^−6^) was identified as marginally significant and functional studies in auditory cells indicated that knockdown of *OTOS* was associated with higher cellular sensitivity to cisplatin.^12^ A GWAS of cisplatin‐induced peripheral neuropathy identified no significant SNP associations.^13^

In the present study, we evaluated 1680 TCS from the Platinum Study to comprehensively investigate and quantify the extent to which nongenetic associations, including modifiable comorbidities (hypertension, hypercholesterolemia, tobacco use, excess alcohol consumption, and loud noise exposure) were related to all three neurotoxicities (hearing loss, tinnitus, and peripheral neuropathy). To identify novel genetic signals associated with cisplatin‐induced hearing loss, tinnitus, and peripheral neuropathy, we performed GWAS in over 1000 survivors. We also attempted to replicate previously identified SNP associations for each phenotype.

## 2 MATERIALS AND METHODS

### 2.1 Patients and study design

All 1680 patients were enrolled in the Platinum Study, which includes eight cancer centers in the United States, Canada, and United Kingdom.^7^, ^8^ Eligibility criteria are illustrated in Figure S1 and as previously described.^11^, ^12^, ^13^ All survivors provided written consent for study participation, access to medical records, and genotyping. Study procedures were approved by each institution's Human Subject Review Board and conducted in accordance with the U.S. Common Rule. The overall study design and sample size for each analysis (GWAS and independent SNP analysis) are also shown in Figure S1.

### 2.2 Assessments

Patient data were determined at clinical follow‐up or collected from medical records following a standardized protocol as previously described.^8^ Data collected from medical records included: treatment regimen with cumulative dose and the number of cycles of each chemotherapeutic, and age at diagnosis of TC. Data collected during a physical examination included: age, weight, and height. At this clinical evaluation, the patient also completed a validated self‐reported questionnaire, and blood was collected for genotyping. In addition, pure‐tone air conduction thresholds were measured bilaterally at the frequency range 0.25–12 kHz to quantify hearing.

Information regarding hypertension, hypercholesterolemia, persistent dizziness/vertigo, overall health conditions, noise exposure, alcohol consumption, and tobacco use were also obtained from these questionnaires as previously described (Methods S1).^11^, ^12^, ^13^

### 2.3 Establishment of the cisplatin‐induced hearing loss, tinnitus and peripheral sensory neuropathy phenotypes

Cisplatin‐induced hearing loss was modeled as a quantitative phenotype using the geometric mean of bilateral average air conduction thresholds measured at frequencies between 4 and12 kHz as described previously (*n* = 1258; previous study analyzed 488 subjects of this cohort^11^).

Cisplatin‐induced tinnitus was defined as previously described (*n* = 1217, previous study analyzed 762 subjects of this cohort^12^), based on response to the question, “Have you had in the last 4 weeks: ringing or buzzing in the ears?” from the validated SCIN questionnaire.^15^ Answers included: not at all, a little, quite a bit, very much. Survivors were dichotomized to tinnitus case/control groups. Controls were survivors who responded “not at all.” Cases were only survivors who responded “quite a bit” or “very much” with the exclusion of those who answered “a little” to establish a more rigorous phenotype. In a separate question, survivors were asked, “Do you have ringing or buzzing in the ears?” For consistency, tinnitus cases responding “no” to this question, but “quite a bit” or “very much” to the SCIN question were excluded from the analysis.

For cisplatin‐induced peripheral neuropathy, the frequency of sensory neuropathy was evaluated using eight items in the validated EORTC‐CIPN20 (Table S1).^16^ We converted sample responses to a 0–3 numeric scale: 0 for “none”, 1 for “a little”, 2 for “quite a bit”, 3 for “very much.” We created four categories to represent the severity of peripheral neuropathy for 1653 TCS using a summary statistic mathematically equivalent to the standard scoring algorithm^17^ and combined groups 2 and 3 due to small sample sizes as described in our previous study that analyzed 680 subjects of this cohort.^13^

### 2.4 Analysis of phenotypes with patient characteristics

Associations between survivors' characteristics and cisplatin‐induced hearing loss, tinnitus, and peripheral neuropathy were evaluated using linear, logistic, and proportional odds ordinal logistic regression, respectively. Models were adjusted for age at clinical examination and cumulative cisplatin dose, which were used as continuous variables.

To investigate cumulative cisplatin dose threshold, regression analyses were also conducted adjusting for age at clinical examination for each phenotype with cumulative cisplatin dose as a categorical variable (<300, 300, 400, >400 mg/m^2^). Survivors receiving >300 and < 400 mg/m^2^ cumulative cisplatin dose were excluded due to small sample size (*n* = 69). In addition, the majority of TCSs receive typical homogeneous treatments with cumulative cisplatin dose of 300 or 400 mg/m^2^. We further performed a two‐proportion *z*‐test for each phenotype, comparing the proportion of survivors with the phenotype. For cisplatin‐induced hearing loss, the two‐proportion *z*‐test was applied on patient proportions with hearing threshold >20 and ≤20 dB; for tinnitus, the population proportions of the case and control were compared; and the proportion of patients with severe cisplatin‐induced peripheral neuropathy was compared to the proportion of the remaining cohort.

All tests were two‐sided at a significance threshold of *p* <0.05. All statistical analyses were performed in R 3.6.1 and plotted with ggplot2 unless otherwise specified.

### 2.5 Genotyping and imputation

At the time of clinical evaluation, DNA was extracted from peripheral blood of survivors. Genotyping was performed on the Infinium Global Screening Array‐24 chip (GSA‐24v1‐0_A1; Illumina) at Regeneron Pharmaceuticals. Sample‐level and SNP‐level quality control criteria were consistent with our previous studies^11^, ^12^, ^13^ and illustrated in Figures S2–S4.The minor allele frequency (MAF) threshold was set to 0.01 for tinnitus and 0.05 for hearing loss and peripheral neuropathy. Imputation was done on the University of Michigan Imputation Server. SNPs and samples passing QC criteria comprised the input set for imputation with EAGLE phasing using the Haplotype Reference Consortium.^18^, ^19^, ^20^ SNPs with imputation *R* ^2^ < 0.8, MAF < MAF threshold, HWE *p* < 1 × 10^6^, and INFO scores > 1.05 or <0.6 were excluded (Figures S2–S4).

### 2.6 Genome‐wide analyses

**FIGURE 1:**
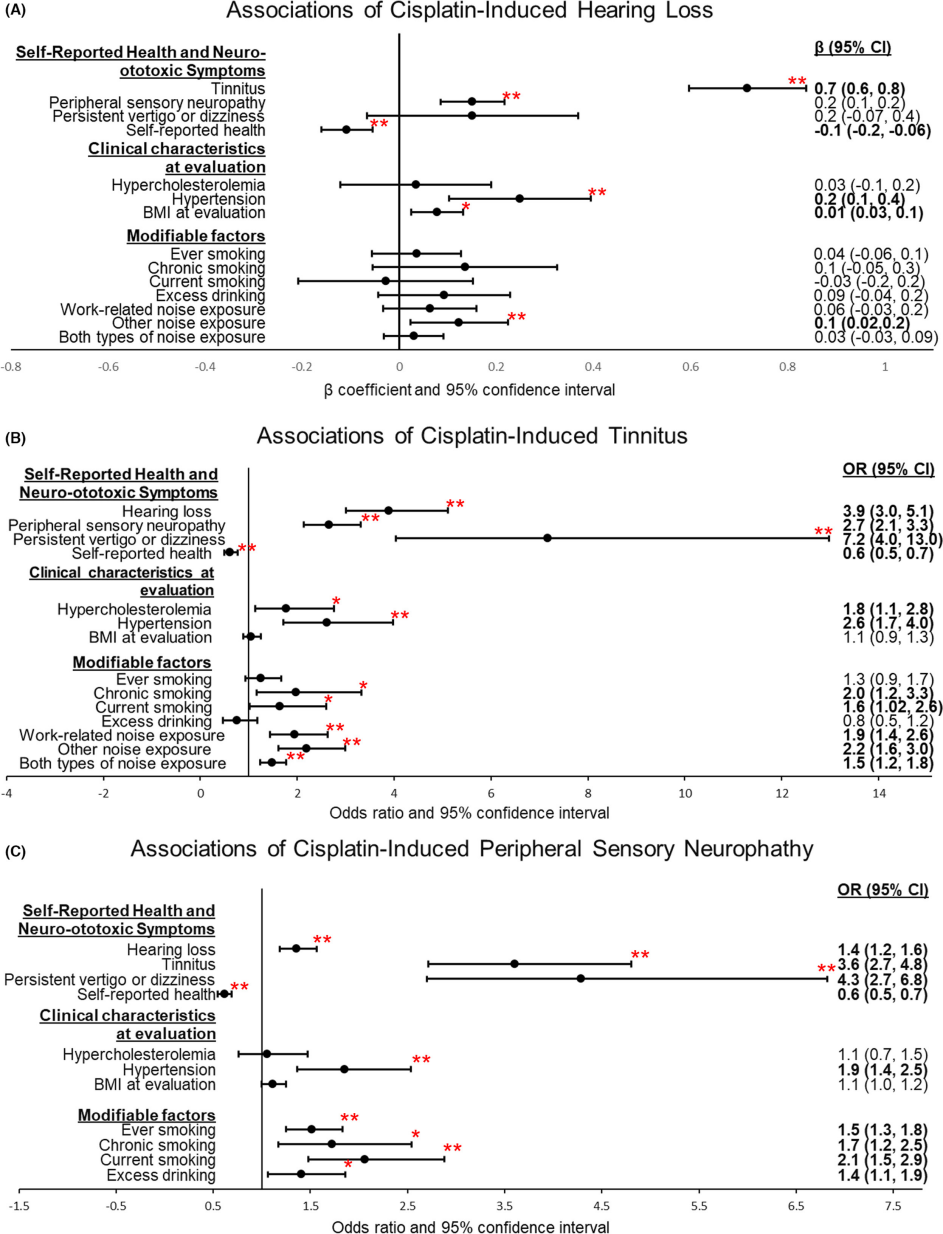
Associations between survivor characteristics and cisplatin‐induced neurotoxicities. Forest plots of regression coefficients and 95% confidence interval (95% CI) for: (A) cisplatin‐induced hearing loss, (B) cisplatin‐induced tinnitus, (C) cisplatin‐induced peripheral sensory neuropathy. All models were adjusted for age at clinical evaluation and cumulative cisplatin dosage. Bolded regression coefficients (95% CI) are significantly associated at *α* = 0.05. *p ≤ 0.05; **p ≤ 0.001

All GWAS were conducted using methodology similar to previous studies,^11^, ^12^, ^13^ with significantly more samples and use of a different Illumina genotyping array. GWAS for hearing loss was done with linear regression for 1071 survivors with 5,385,324 SNPs using cumulative cisplatin dose, age at clinical examination, and the first 10 genetic principal components as covariates.^11^ GWAS for tinnitus was performed with logistic regression on 1037 TCS and 7,657,611 SNPs using cumulative cisplatin dose, age at diagnosis, the first five genetic principal components as covariates as described previously.^12^ We also adjusted for overall noise exposure in the tinnitus GWAS, because noise is a known risk factor for tinnitus^21^ and because regression analyses from the current study demonstrated strong association between tinnitus and noise exposure (noise exposure at work: OR = 1.9, 95% CI = 1.4–2.6, *p* < 0.0001; outside of work: OR = 2.2, 95% CI = 1.6–3.0, *p* < 0.0001; both at work and outside of work: OR = 1.5, 95% CI = 1.2–1.8, *p* < 0.0001; Figure 1). GWAS for tinnitus and hearing loss was done in PLINK v1.9. Using age at diagnosis and the first 10 European genetic principal components as covariates, GWAS for peripheral neuropathy was performed for 1397 survivors with 4,875,644 SNPs by ordinal logistic regression in R 3.6.1 with the MASS package.^22^ All GWAS assumed additive effects and had a genome‐wide significance threshold set to *p* < 5 × 10^−8^.

For the gene‐based association analysis, the aggregated effect of all SNPs within a gene was analyzed simultaneously in the functional mapping and annotation of GWAS (FUMA) platform using the multi‐marker analysis of genomic annotation (MAGMA) method that is based on a multiple regression model that efficiently incorporates linkage disequilibrium between SNPs.^23^, ^24^ Summary statistics were then uploaded to FUMA for gene‐based association analysis and for region‐based plotting. SNPs were mapped to 18,544, 18,819, 18,106 protein coding genes for hearing loss, tinnitus, and peripheral neuropathy respectively, producing a significance threshold of 2.7 × 10^−6^. After collecting the candidate genes that were genome‐wide significant or nearly significant, we extracted SNPs with a GWAS *p* < 1 × 10^−5^ and within 25 kb upstream or downstream of these genes. We then searched these SNPs in GTEx portal for splicing quantitative trait loci (QTL) or expression QTLs without specifying tissue types, but associated with the candidate genes.

### 2.7 Replication of candidate SNPs

Using independent cohorts, we evaluated two SNPs previously associated with cisplatin‐induced neurotoxicities: rs62283056 in *WFS1* for hearing loss^11^ and rs7606353 in *OTOS* for tinnitus.^12^ We performed independent SNP association tests adjusting with covariates using linear regression and assuming linear additive SNP effect. Excluding participants in the previous studies, linear regression, and logistic regression were performed on completely independent replication cohorts for hearing loss (*n* = 606) and tinnitus (*n* = 325), respectively. The covariates were consistent with the original GWAS analysis, and the significance threshold is 0.05.

### 2.8 Evaluation of cisplatin sensitivity based on gene expression in silico

Gene expression data for two genes (TXNRD1, WNT8) from the FUMA gene‐based analysis in central nervous system (CNS) was acquired from the Cancer Cell Line Encyclopedia.^25^ Cisplatin sensitivity, which was measured as the area under the dose–response curve, was obtained from the Genomics of Drug Sensitivity in Cancer Project.^26^ We then performed Spearman correlation and linear regression to analyze the associations between gene expression and drug sensitivity of cancer cell lines. Genes with missing expression data were excluded. There were only four non‐missing gene expression values in CNS tumor cell lines from the Cancer Cell Line Encyclopedia^25^ available for WNT8, preventing an analysis of gene expression and cell sensitivity to cisplatin. These analyses were done in R 3.6.1.

## 3 RESULTS

### 3.1 Cohort characteristics

Cohort characteristics stratified by each cisplatin‐induced phenotype, hearing loss, tinnitus, and peripheral neuropathy are provided in Table 1 and Table S2. Overall median age at diagnosis and age at clinical evaluation were 30 (range: 10–60 years and 37 (range: 18–75) years, respectively. With an overall time since therapy completion of 4 (range: 0–37) years; all neurotoxicities were long‐term toxicities. Overall median BMI at clinical evaluation was 27 (range:18–67) kg/m^2^. Most survivors were treated with bleomycin, etoposide, and cisplatin (BEP; 54.4%) or etoposide and cisplatin (EP; 37.5%). 46.5% of survivors received <400 mg/m^2^ cumulative cisplatin dose, and 53.5% of survivors were treated with ≥400 mg/m^2^.

**TABLE 1:** Demographic features, clinical characteristics, and patient‐reported outcomes for 1258, 1217, and 1653 male germ cell tumor survivors included in studies for cisplatin‐induced hearing loss, tinnitus, and peripheral neuropathy

Characteristic	Hearing loss^a^	Tinnitus^b^			Peripheral sensory neuropathy^c^			
		All survivors	No (Controls)	Yes (Cases)	All survivors	None	Mild	Severe
*n*	1258	1217	979	238	1653	704	740	209
Age at last observation, year, Median (range)	37 (18–74)	37 (18–75)	36 (18–75)	40 (18–74)	37 (18–75)	34 (18–72)	38 (18–75)	41 (20–65)
Age at testicular cancer diagnosis, year, Median (range)	31 (10–60)	30 (10–60)	30 (10–60)	32 (10–55)	30 (10–60)	28 (10–54)	32 (10–60)	34 (13–55)
Time since therapy completion, year, Median (range)	4 (0–37)	4 (0–37)	4 (0–37)	4 (0–35)	4 (0–37)	4 (0–37)	4 (0–35)	4 (0–37)
BMI at evaluation, kg/m^2^, Median (range)	27 (18–67)	27 (18–67)	27 (18–60)	28 (18–67)	27 (18–67)	27 (18–66)	27 (18–54)	29 (18–67)
Hearing thresholds, dB, Median (range)^a^	18 (1–96)	18 (1–96)	15 (1–93)	38 (2–96)	18 (1–96)	15 (1–93)	20 (2–94)	28 (1–96)
*Chemotherapy regimen* ^d^								
BEP	696 (55.3)	662 (54.5)	534 (54.7)	128 (53.8)	897 (54.4)	370 (52.7)	431 (58.2)	96 (45.9)
EP	458 (36.4)	462 (38.0)	372 (38.1)	90 (37.8)	623 (37.7)	271 (38.6)	261 (35.3)	91 (43.5)
VIP	30 (2.4)	20 (1.6)	13 (1.3)	7 (2.9)	35 (2.1)	12 (1.7)	17 (2.3)	6 (2.9)
VeIP	1 (0.1)	2 (0.2)	2 (0.2)	0 (0.0)	2 (0.1)	2 (0.3)	0 (0.0)	0 (0.0)
PVB	3 (0.2)	2 (0.2)	1 (0.1)	1 (0.4)	4 (0.2)	1 (0.1)	2 (0.3)	1 (0.5)
Other	70 (5.6)	67 (5.5)	55 (5.6)	12 (5.0)	90 (5.5)	46 (6.5)	29 (3.9)	15 (7.2)
*Cumulative dose of cisplatin, mg/m* ^*2*^ ^e^								
Median(range)	400 (100–1000)	400 (100–1000)	400 (130–1000)	400 (100–800)	400 (100–1000)	400 (190–600)
<300	59 (4.7)	82 (6.8)	68 (7.0)	14 (5.9)	102 (6.2)	41 (5.9)	50 (6.8)	11 (5.3)
300	468 (37.4)	440 (36.5)	366 (37.8)	74 (31.1)	592 (36.1)	272 (39.1)	271 (36.7)	49 (23.5)
>300 and < 400	50 (4.0)	42 (3.5)	31 (3.2)	11 (4.6)	66 (4.0)	23 (3.3)	36 (4.9)	7 (3.4)
400	618 (49.5)	579 (48.0)	467 (48.3)	112 (47.1)	793 (48.3)	318 (45.8)	350 (47.4)	125 (60.1)
>400	55 (4.4)	63 (5.2)	36 (3.7)	27 (11.3)	88 (5.4)	41 (5.9)	31 (4.2)	16 (7.7)
*Persistent dizziness/vertigo* ^f^								
Yes	53 (4.5)	53 (4.6)	23 (2.4)	30 (14.4)	71 (4.6)	12 (1.8)	35 (5.1)	24 (12.8)
No	1125 (95.5)	1104 (95.4	926 (97.6)	178 (85.6)	1487 (95.4)	665 (98.2)	658 (94.9)	164 (87.2)
*Self‐reported health* ^g^								
Excellent	210 (16.9)	194 (16.0)	172 (17.7)	22 (9.3)	268 (16.3)	145 (20.7)	107 (14.5)	16 (7.7)
Very good	524 (42.2)	502 (41.4)	432 (44.4)	70 (29.4)	670 (40.7)	313 (44.7)	306 (41.4)	51 (24.5)
Good	419 (33.7)	421 (34.8)	315 (32.3)	106 (44.5)	582 (35.3)	214 (30.6)	269 (36.4)	99 (47.6)
Poor/fair	90 (7.2)	95 (7.8)	55 (5.6)	40 (16.8)	127 (7.7)	28 (4.0)	57 (7.7)	42 (20.2)
*Hypertension and on medication* ^h^								
Yes	142 (11.8)	136 (11.7)	85 (9.1)	51 (22.8)	189 (12.0)	47 (6.9)	96 (13.7)	46 (23.6)
No	1057 (88.2)	1023 (88.3)	850 (90.9)	173 (77.2)	1390 (88.0)	634 (93.1)	607 (86.3)	149 (76.4)
*Hypercholesterolemia and on medication* ^i^								
Yes	131 (10.9)	131 (11.2)	90 (9.5)	41 (18.5)	166 (10.4)	55 (7.9)	81 (11.4)	30 (15.6)
No	1072 (89.1)	1040 (88.8)	859 (90.5)	181 (81.5)	1426 (89.6)	637 (92.1)	627 (88.6)	162 (84.4)

*Note*: Data presented as number (%) unless otherwise noted.

Abbreviations: BMI, body mass index; BEP, bleomycin, etoposide, and cisplatin; EP, etoposide and cisplatin; VIP, cisplatin, etoposide, and ifosfamide; VeIP, cisplatin, vinblastine, and ifosfamide; PVB, cisplatin, bleomycin, and maintenance vinblastine.

^a^ One thousand two hundred and fifty‐eight patients were included with quantitative values modeled using the geometric mean of bilateral average air conduction thresholds measured at frequencies between 4 and 12 kHz as described previously.^10^, ^11^

^b^ Tinnitus phenotype excludes 463 participants who did not answer‐related questions. Cases are restricted to survivors who reported “quite a bit” or “very much” tinnitus. Survivors who reported “a little” tinnitus (*n* = 426) are excluded from the table and all analyses.

^c^ Following conversion of the Likert scale: “none, a little, quite a bit, very much” to a 0–3 numeric scale, we created four categories to represent the severity of peripheral neuropathy using a summary statistic and combined groups 2 and 3.^13^ Phenotype excludes 27 participants for whom the variables were not stated.

^d^ BEP category includes survivors who received only bleomycin, etoposide, and cisplatin; EP includes survivors who received only etoposide and cisplatin. VIP includes survivors who received only cisplatin, etoposide, and ifosfamide; VeIP includes survivors who received only cisplatin, vinblastine, and ifosfamide; PVB includes survivors who received only cisplatin, bleomycin, and maintenance vinblastine. Both tinnitus and peripheral sensory neuropathy had two survivors with missing dose data.

^e^ Category excludes eight participants with incomplete dose data for hearing loss, 11 for tinnitus, and 12 for peripheral neuropathy.

^f^ Persistent vertigo or dizziness status was not stated for 80 hearing loss participants, 60 tinnitus participants, and 95 peripheral sensory neuropathy participants.

^g^ Self‐reported health status was not stated for 15 hearing loss participants, five tinnitus participants, and six peripheral sensory neuropathy participants.

^h^ Hypertension status was not stated for 59 participants with hearing loss, 58 participants with tinnitus, and 74 participants with peripheral sensory neuropathy.

### 3.2 Interdependence of cisplatin‐induced hearing loss, tinnitus, peripheral neuropathy

Cisplatin‐induced hearing loss, tinnitus, and peripheral neuropathy were interdependent, adjusting for age and cumulative cisplatin dose (*p* < 0.0001). Hearing loss was positively correlated with tinnitus (*β* = 0.7, 95% CI = 0.6–0.8) and peripheral neuropathy (*β* = 0.2, 95% CI = 0.1–0.2). Risk of tinnitus was 3.9‐fold (95% CI = 3.0–5.1) and 2.7‐fold (95% CI = 2.1–3.3) greater for survivors with more severe hearing loss and peripheral neuropathy, respectively. Similar associations were observed for peripheral neuropathy (hearing loss: OR = 1.4, 95% CI = 1.1–1.6; tinnitus: OR = 3.6, 95% CI = 2.7–4.8).

### 3.3 Associations with cisplatin‐induced hearing loss

We identified a negative correlation of hearing loss with self‐reported health (poor‐excellent; *β* = −0.1, 95% CI = −0.2 to −0.06, *p* < 0.0001). Hypertension (*β* = 0.2, 95% CI = 0.1–0.4, *p* = 8.5 × 10^−4^), BMI at evaluation (*β* = 0.01, 95% CI = 0.03–0.1, *p* = 0.004), and nonwork‐related noise exposure (*β* = 0.1, 95% CI = 0.02–0.2, *p* = 0.02) were positively associated with hearing loss. However, hearing loss was not associated with persistent vertigo, hypercholesterolemia, smoking status, excess alcohol, work‐related or both types of noise exposure (Figure 1).

### 3.4 Associations with cisplatin‐induced tinnitus

Self‐reported health was significantly poorer in cases than in controls (OR = 0.6, 95% CI = 0.5–0.7, *p* <0.0001). Tinnitus was positively correlated with persistent vertigo (OR = 7.2, 95% CI = 4.0–13.0, *p* < 0.0001), hypertension (OR = 2.6, 95% CI = 1.7–4.0, *p* < 0.0001) and hypercholesterolemia (OR = 1.8, 95% CI = 1.1–2.8, *p* = 0.01). We also observed positive associations between tinnitus and chronic smoking (OR = 2.0, 95% CI = 1.2–3.3, *p* = 0.01), current smoking (OR = 1.6, 95% CI = 1.02–2.6, *p* = 0.04), and noise exposure (at work: OR = 1.9, 95% CI = 1.4–2.6, *p* <0.0001, outside of work: OR = 2.2, 95% CI = 1.6–3.0, *p* < 0.0001, and the combination of both types of noise exposure: OR = 1.5, 95% CI = 1.2–1.8, *p* < 0.0001). However, no association was observed between tinnitus and ever smoking, excess alcohol consumption, or BMI at clinical evaluation (Figure 1).

### 3.5 Association with cisplatin‐induced peripheral neuropathy

We identified a strong negative correlation between peripheral neuropathy and self‐reported health (OR = 0.6, 95% CI = 0.5–0.7, *p* < 0.0001). Persistent vertigo (OR = 4.3, 95% CI = 2.7–6.8, *p* < 0.0001) and hypertension (OR = 1.9, 95% CI = 1.4–2.5, *p* < 0.0001) were positively associated with peripheral neuropathy. Risk of peripheral neuropathy was also positively associated with smoking status (ever smoking: OR = 1.5, 95% CI = 1.3–1.8, *p* < 0.0001; chronic smoking: OR = 1.7, 95% CI = 1.2–2.5, *p* = 0.006, current smoking: OR = 2.1, 95% CI = 1.5–2.9, *p* < 0.0001) and excess alcohol consumption (OR = 1.4, 95% CI = 1.1–1.9, *p* = 0.02). BMI at evaluation was marginally significantly associated with peripheral neuropathy (OR = 1.1, 95% CI = 1.0–1.2, *p* = 0.06), but no association was found between peripheral neuropathy and hypercholesterolemia (Figure 1).

### 3.6 Effect of cumulative cisplatin dose on cisplatin‐induced hearing loss, tinnitus, and peripheral neuropathy

**FIGURE 2:**
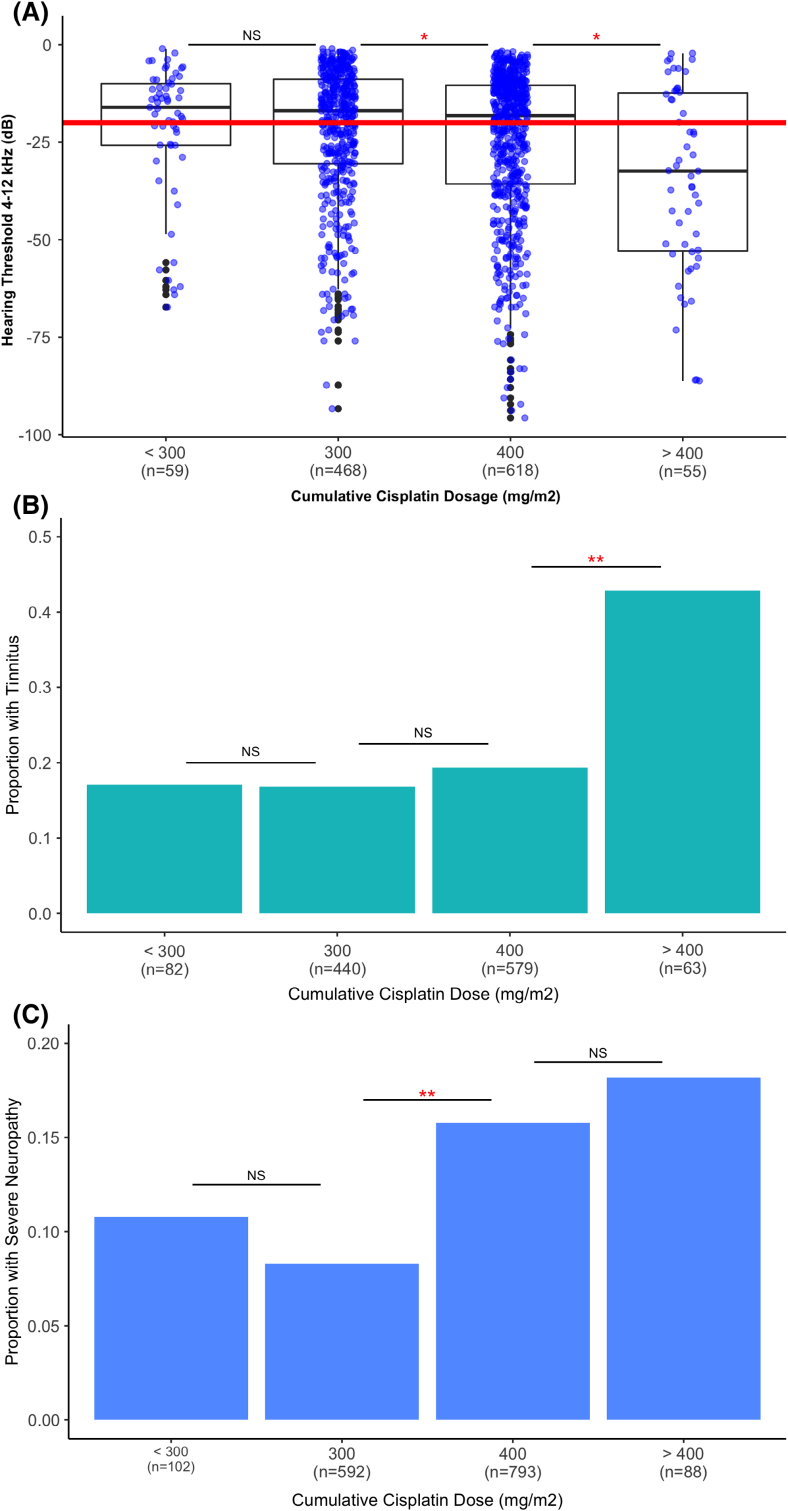
Effect of cumulative cisplatin dose on cisplatin‐induced tinnitus, hearing loss, and peripheral sensory neuropathy. (A) Boxplot showing hearing thresholds by cumulative cisplatin dose group (<300, 300, 400, and >400 mg/m^2^) illustrates significantly increased risk of hearing loss in patients treated with doses 400 mg/m^2^ compared to 300 and >400 mg/m^2^ compared to 400 mg/m^2^; (B) bar plot showing the frequency of tinnitus by cumulative cisplatin dose group demonstrated significantly increased risk of tinnitus in doses >400 mg/m^2^‐treated‐survivors compared to 400 mg/m^2^‐treated‐survivors. (C) Bar plot showing the frequency of severe peripheral neuropathy by cumulative cisplatin dose group illustrates significantly increased risk of peripheral neuropathy in patients treated with doses 400 mg/m^2^ compared to 300 mg/m^2^‐treated‐survivors. The number of subjects per category is presented on the *x* axis under the dose group label. *p < 0.05; **p < 0.005

Risk of more severe hearing loss for survivors who received 400 mg/m^2^ was greater compared with those receiving 300 mg/m^2^ (*β* = 0.1, 95% CI = 0.01–0.2, *p* = 0.02); a stronger increase of risk was found between 400 mg/m^2^‐treated‐TCS and >400 mg/m^2^‐treated‐TCS (*β* = 0.3, 95% CI = 0.06–0.5, *p* = 0.01; Figure 2A). Survivors who received >400 mg/m^2^ were 3.1‐fold more likely to have tinnitus (95% CI: 1.8–5.3‐fold, *p* < 0.0001) compared with those receiving 400 mg/m^2^ (Figure 2B) with no significant differences in tinnitus risk for survivors treated with <300 mg/m^2^ versus 300 mg/m^2^ or for 300 mg/m^2^ versus 400 mg/m^2^ cisplatin. Performing the same analysis for peripheral neuropathy, survivors who were treated with 400 mg/m^2^ were at 1.4‐fold increased risk compared with those receiving 300 mg/m^2^ (95% CI: 1.2–1.7‐fold, *p* = 0.003) with no significant differences for <300 mg/m^2^ versus 300 mg/m^2^ and 400 mg/m^2^ versus >400 mg/m^2^ (Figure 2C).

To further confirm the dose threshold of cisplatin‐induced neurotoxicities, two‐proportion *z*‐tests were performed to compare patient proportions in dose groups. We only compared dose groups that demonstrated significant difference in neurotoxicity risks according to the regression analyses described above. The proportion of survivors with hearing loss and tinnitus was significantly greater following treatment with >400 mg/m^2^ compared to 400 mg/m^2^ cumulative cisplatin dose (hearing loss: 65% vs. 46%, *p* = 0.01; tinnitus: 43% vs. 19%, *p* < 0.0001). Although proportions of survivors with hearing loss treated with 400 mg/m^2^ and 300 mg/m^2^ were not significantly different, significant proportional differences were observed comparing survivors with peripheral neuropathy (16% vs. 8%, *p* < 0.0001).

### 3.7 Genome‐wide association studies of cisplatin‐induced neurotoxicities

#### 3.7.1 GWAS of cisplatin‐induced hearing loss

**FIGURE 3:**
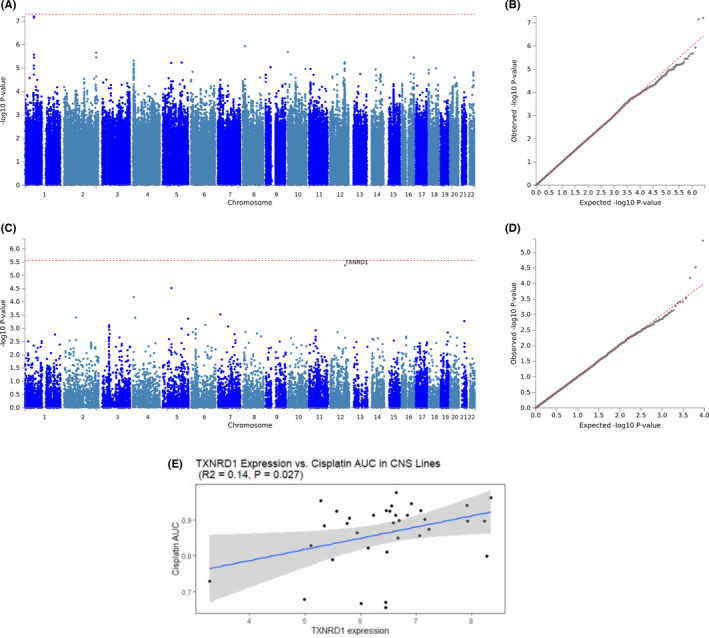
Genome‐wide single nucleotide polymorphism (SNP) and gene‐based association studies of cisplatin‐induced hearing loss. (A) Manhattan plot of genome‐wide association study (GWAS) results for cisplatin‐induced hearing loss. (B) Quantile–Quantile plot of GWAS results for cisplatin‐induced hearing loss. Covariates in the GWAS include cumulative cisplatin dose, age at clinical evaluation, and 10 European genetic principal components accounting for population substructure. (C) Manhattan plot of the gene‐based association analysis identifies *TXNRD1* (*p* = 4.2 × 10^−6^) as nearly genome‐wide significant. Summary statistics for SNP‐based GWAS were uploaded to functional mapping and annotation to run a gene‐based association analysis based on a multiple linear principal components regression to determine the aggregated effect of all SNPs within a gene. Inputted SNPs were mapped to 18,544 protein coding genes, producing a significance threshold of *p* = 0.05/18,544 (2.7 × 10^−6^). (D) Quantile–Quantile plot of results from the gene‐based association analysis. (E) Scatter plots of cisplatin sensitivity as a function of normalized *TXNRD1* expression in central nervous system (CNS) tumor cell lines (*ρ* = 0.4, *p* = 0.04; *R* ^2^ = 0.1, *p* = 0.03). Cisplatin sensitivity, measured as the area under the cisplatin dose–response curve, for all CNS tumor cell lines extracted from CancerRX. Gene expression data were downloaded from the Cancer Cell Line Encyclopedia. Expression data were rank normalized to fit a normal distribution prior to analysis. Correlation was assessed nonparametrically using the Spearman rank method, as well as by linear regression

In a GWAS of hearing loss for 1071 TCS, no SNP met genome‐wide significance (Table S3); however, *TXNRD1* was nearly genome‐wide significant for the gene‐based analysis (*p* = 4.2 × 10^−6^; Figure 3A–D). In addition, expression levels of *TXNRD1* were positively correlated with cisplatin resistance in CNS cell lines (Spearman *ρ* = 0.4, *p* = 0.04; *R* ^2^ = 0.1, *p* = 0.03; Figure 3E), indicating that high gene expression of *TXNRD1* is associated with cellular resistance to cisplatin. To examine the specificity of this correlation with cisplatin, we evaluated the relationship of seven other antineoplastic agents (5‐fluorouracil, bleomycin, bortezomib, docetaxel, etoposide, and vinblastine) and *TXNRD1* expression in CNS tumor cell lines, yet no other significant relationships were observed Table S4. Further, a SNP on chromosome 12, rs4406890, located in the intronic region of *TXNRD1* was borderline significant with a *p*‐value of 5.7 × 10^−6^. This intronic variant is a sQTL that regulates alternative splicing of *TXNRD1* in skeletal muscle as reported by the GTEx Portal.

#### 3.7.2 GWAS of cisplatin‐induced tinnitus

**FIGURE 4:**
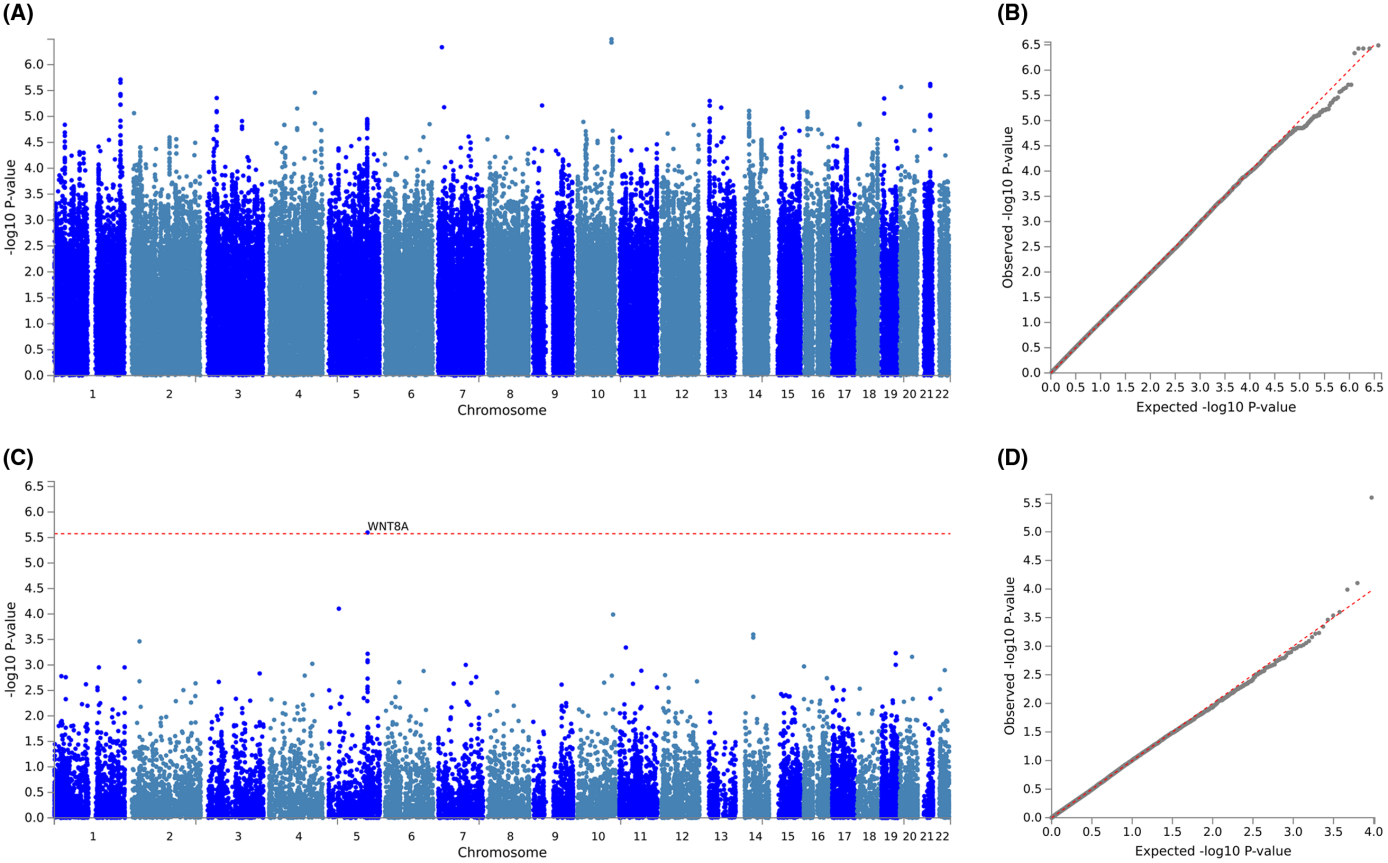
Genome‐wide single nucleotide polymorphism (SNP) and gene‐based association studies of cisplatin‐induced tinnitus. (A) Manhattan plot of genome‐wide association study (GWAS) results for cisplatin‐induced tinnitus. (B) Quantile–Quantile plot of GWAS results for cisplatin‐induced tinnitus. Covariates in the GWAS include cumulative cisplatin dose, noise exposure, age at clinical evaluation, and five European genetic principal components accounting for population substructure. (C) Manhattan plot of the gene‐based association analysis identifies *WNT8A* (*p* = 2.5 × 10^−6^) as genome‐wide significant. Summary statistics for SNP‐based GWAS were uploaded to functional mapping and annotation to run a gene‐based association analysis based on a multiple linear principal components regression to determine the aggregated effect of all SNPs within a gene. Inputted SNPs were mapped to 18,819 protein coding genes, producing a significance threshold of *p* = 0.05/18,819 (2.7 × 10^−6^). (D) Quantile–Quantile plot of results from the gene‐based association analysis

There were 979 (59.6%), 426 (25.9%), 118 (7.2%), and 120 (7.3%) survivors reporting none, mild, moderate, and severe tinnitus, respectively. After removing survivors who reported mild tinnitus, subjects were then dichotomized to 979 (80.4%) controls (none) and 238 (19.6%) cases (moderate/severe). No SNPs met genome‐wide significance in the GWAS of tinnitus (Figure 4; Table S5); however, gene‐based association analysis identified *WNT8A* as genome‐wide significant (*p* = 2.5 × 10^−6^).

#### 3.7.3 GWAS of cisplatin‐induced peripheral neuropathy

**FIGURE 5:**
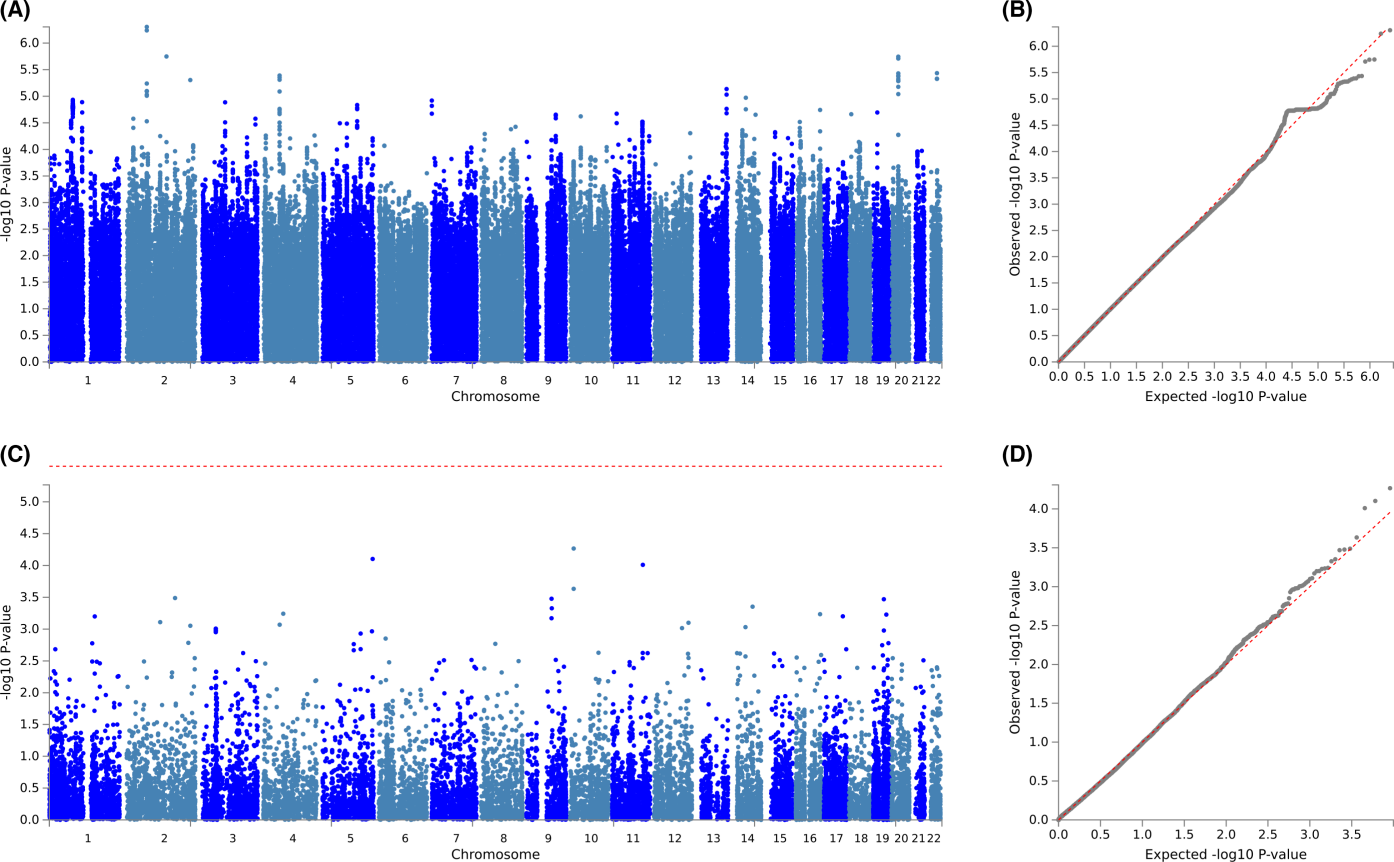
Genome‐wide single nucleotide polymorphism (SNP) and gene‐based association studies of cisplatin‐induced peripheral sensory neuropathy (A) Manhattan plot of genome‐wide association study (GWAS) results for cisplatin‐induced peripheral sensory neuropathy. (B) Quantile–Quantile plot of GWAS results for cisplatin‐induced peripheral sensory neuropathy. Covariates in both GWAS include age at diagnosis and 10 European genetic principal components accounting for population substructure. (C) Manhattan plot of the gene‐based association analysis identifies no genome‐wide significant genes. Summary statistics for SNP‐based GWAS were uploaded to functional mapping and annotation to run a gene‐based association analysis based on a multiple linear principal components regression to determine the aggregated effect of all SNPs within a gene. Inputted SNPs were mapped to 18,106 protein coding genes, producing a significance threshold of *p* = 0.05/18,106 (2.8 × 10^−6^). (D) Quantile–Quantile plot of results from the gene‐based association analysis

Of the 1653 TCS, 704 (42.6%) reported no peripheral neuropathy, 740 (44.8%) reported mild peripheral neuropathy, and 209 (12.6%) reported severe peripheral neuropathy. GWAS was performed on 1397 survivors. Neither the SNP‐based nor gene‐based analysis of peripheral neuropathy identified significant signals (Figure 5; Table S6).

### 3.8 Replication of candidate SNPs

Performing individual SNP association analysis on completely independent cohorts by excluding survivors in previous studies,^11^, ^12^ the association between hearing loss and rs62283056 in *WFS1* was borderline significant (*p* = 0.06). The overall hearing threshold distributions did not differ between discovery and replication cohorts (Table S2), however when analyzing‐specific genotypes, we observed lower median hearing threshold for survivors carrying the risk allele in the replication set (Median: 15.4 dB; Range: 3.1–73.7 dB) compared to the discovery set (Median: 36.0 dB; Range: 6.8–86.9 dB; Figure S5).^11^ This observation is also a potential explanation of the difference in association effect direction (*β*_replication =_ − 0.34, *β*_discovery_ = 0.11). No association (*p* = 0.7) was observed between tinnitus and rs7606353 in *OTOS* (Table S7).^12^

## 4 DISCUSSION

Based on the largest study to date of cisplatin‐induced neurotoxicities, we demonstrate that a substantial proportion of TCS treated with cisplatin‐based chemotherapy experience hearing loss, tinnitus, and peripheral neuropathy, and those that do experience neurotoxicities were more likely to have hypertension and describe their health as poor. Hearing loss, but not tinnitus or periperhal neuropathy, was associated with survivors with greater BMI. Conversely, tinnitus and peripheral neuropathy, but not hearing loss, was associated with persistent vertigo or dizziness. Similar results were observed in evaluating modifiable risk factors demonstrating survivors who are either chronic or current smokers are more likely to experience tinnitus or peripheral neuropathy, but not hearing loss. We identified a marked increase in risk for peripheral neuropathy (1.3‐fold) and significantly more hearing loss when comparing 300 mg/m^2^ against 400 mg/m^2^ cisplatin dose. In contrast, there is no significant difference for the risk of tinnitus, yet there is a significant risk (3.1‐fold) above 400 mg/m^2^ compared to 400 mg/m^2^. In addition, gene‐based association analysis identified *WNT8A* significantly associated with tinnitus.

### 4.1 Genetic findings for cisplatin‐induced tinnitus and hearing loss

For the first time, we identified *WNT8A* to be genome‐wide significant for tinnitus and *TXNRD1* as having borderline genome‐wide significance for hearing loss following cisplatin treatment through a gene‐based association analysis. Human *WNT8A* mRNA is expressed in NT2 cells with neuronal differentiation potential^27^ and was reported to be involved in the development of early embryos as well as germ cell tumors through activation of the WNT β‐catenin‐TCF pathway.^28^ Mattes et al.^29^ have illustrated that *WNT8A* plays a key role in the Wnt/β‐catenin signaling pathway, inducing cell proliferation in a variety of cancer types, including colorectal cancer, pancreatic cancer, and gastric cancer. Wnt8a (mouse homolog) is expressed in the hindbrain and is involved in early inner ear development in mice.^30^ Using the gEAR database, *Wnt8a* and *Txnrd1* are expressed in mouse cochlea.^31^ To inhibit the Wnt signaling pathway, several antineoplastic therapies have been developed, and many agents are currently in early phase oncology clinical trials.^32^ Nevertheless, the role WNT8A in relation to tinnitus and/or de novo tinnitus is not yet established. TXNRD1, associated with cisplatin‐induced hearing loss, is critical for redox regulation, antioxidant defense, and synthesis of deoxyribonucleotides.^33^ Both increasing age and more severe hearing loss in a mouse model are associated with downregulation of the *Txnrd1* gene in the auditory portion of the inner ear and cochlea.^34^

### 4.2 Cisplatin‐induced tinnitus

The association between tinnitus and persistent dizziness/vertigo in our cisplatin‐treated TCS has also been shown for de novo tinnitus, as inner ear problems are strongly implicated in balance disorders, including vertigo.^35^ Both hypertension^36^, ^37^ and hypercholesterolemia^38^, ^39^ have also been demonstrated to be risk factors for de novo tinnitus. Przewoźny et al.,^40^ suggested that hypertension causes damage to the stria vascularis by reducing cochlear oxygen partial pressure and disrupting the recycling of potassium ions in the cochlea. The relationship with hypercholesterolemia may be related to evidence that cholesterol levels can impair cochlear microcirculation^41^ and the function of cochlear outer hair cells.^42^ Accordingly, these findings suggest that lowering abnormally high serum lipid levels and adequate control of hypertension may decrease the likelihood or severity of tinnitus.

### 4.3 Other findings

We observed greater risk of both tinnitus and peripheral neuropathy for chronic smokers and current smokers. Tobacco use is known to be associated with poorer response to cancer treatments, increased cancer mortality, and risk of second primary cancer.^43^ Continued smoking in patients with cancer is associated with significant incremental costs for further cancer treatment when first‐line therapy fails.^44^ In addition, previous investigations also demonstrated significant increases in the neurotoxic effects of chemotherapy in smokers, supporting the findings of the current study.^12^, ^45^ The 2020 Surgeon General's Report^46^ indicates that smoking cessation improves patients' quality of life and adds up to 10 years to their lifespan. In addition, with clear evidence that smoking cessation is beneficial both before and after cancer treatment for many cancer types, this report^46^ emphasized the need to include smoking cessation as a standard part of clinical cancer care.^46^

Cisplatin‐based therapy consists primarily of either three cycles of BEP or four cycles of EP resulting in a cumulative dose of 300 or 400 mg/m^2^ cisplatin, respectively although occasional patients may receive doses of cisplatin greater than 400 mg/m^2^. Our study evaluated the risk of toxicity at these cumulative doses to provide physicians valuable information to make an informed decision since both BEPX3 and EPX4 are considered curative for testicular cancer.^47^ Our observation showed disproportionally increased risks of hearing loss (*β* = 0.4) or tinnitus (3.1‐fold) following >400 mg/m^2^ cumulative cisplatin versus 400 mg/m^2^. A previous study showed that patients treated with >400 mg/m^2^ cumulative cisplatin dose were 2.6‐fold more susceptible to tinnitus than those treated with ≤400 mg/m^2^ ^12^. In addition, Brydøy et al. found that compared with survivors receiving one to four cycles of cisplatin‐based chemotherapy, TCS given five or more cycles had a 2.2‐fold greater incidence of severe hearing impairment and tinnitus.^48^

We identified a marked increase in risk for peripheral neuropathy when comparing 300 mg/m^2^ cumulative cisplatin dose with 400 mg/m^2^ (1.3‐fold). Consistent with our results, previous studies suggest that significant peripheral neuropathy occurs when cumulative cisplatin dose exceeds 300–400 mg/m^2^.^49^ Krarup‐Hansen et al.^50^ conducted clinical and electrophysiological experiments in 16 TCS treated with BEP, to study the primary site of damage of cisplatin‐induced peripheral sensory neuropathy. The authors reported that the amplitudes of sensory nerve action potentials (SNAP) were reduced 50–60% from the feet and fingers for TCS who received >300 mg/m^2^ cisplatin; and the conduction velocities of SNAP were reduced 10%–15% for survivors treated with cumulative doses of 400–700 mg/m^2^ cisplatin compared to patients treated with <300 mg/m^2^. Among 80 women with recurrent ovarian cancer, Van den Bent and colleagues^51^ reported an increased incidence of peripheral neuropathy development with >420 mg/m^2^ cumulative cisplatin dose compared to 300–420 mg/m^2^. After a cumulative cisplatin dose of 600 mg/m^2^, all patients (*n* = 80) developed some degree of peripheral neuropathy with 30%–40% of patients having moderate sensory neuropathy, and 10% having severe and disabling neuropathy. Taken together, these prior reports^49^, ^50^, ^51^ suggest that a dose threshold exists for disproportionally increased risk of peripheral neuropathy after cisplatin‐based chemotherapy.

In comparison with our previous study on 511 survivors where rs62283056 in *WFS1* was significantly (*p* = 1.4 × 10^−8^) associated with hearing loss,^11^ in this study of 606 survivors, the association was of borderline significance. A potential explanation of the lack of statistical significance is that there were more survivors carrying the risk allele that experienced little or no hearing loss in the replication cohort compared to the discovery cohort.^11^ We did not successfully replicate rs7606353 in *OTOS* likely due to small sample size of the replication cohort (*n* = 325). In addition, the MAF (0.032) was lower than that of 0.04 in the previous study (*n* = 762^12^), which constrained the statistical power of the replication analysis. We evaluated the genome‐wide significance of rs7606353 among the entire cohort of 1037 survivors and found the *p*‐value to be 1.5 × 10^−4^.

### 4.4 Strengths and weaknesses

Major strengths of our investigation include the detailed phenotypic data collected in a large cohort of survivors treated with fairly uniform cisplatin‐based chemotherapy. As a result, we were able to better quantify the associations between cisplatin‐induced neurotoxicities and various comorbidities, and also analyze new contributors. Intrinsic limitations of any cross‐sectional study, that also applies here, is the inability to infer causation between comorbidities and phenotypes (cisplatin‐induced neurotoxicities). The definition of loud noise exposure is another limitation, as the participants self‐reported their exposure, and details with regard to noise duration, loudness, or frequency (or timing in relationship to chemotherapy) was not queried. In addition, teasing out whether hearing loss is due to cisplatin, noise exposure or the combination is a challenge. Furthermore, when analyzing the effect of cumulative cisplatin dose on toxicity, the sample size for <300 and >400 mg/m^2^ treatment groups were significantly smaller compared to the other groups. Lastly, this study focused on a European‐only cohort and the results may not be generalized in other population.

### 4.5 Clinical implications

Our findings emphasize the importance of clinical characteristics associated with cisplatin‐induced neurotoxicities. Clinicians should ensure that patients are aware of these iatrogenic toxicities and their associated comorbidities, including hypertension and hypercholesterolemia. Associated clinical conditions and behavioral characteristics should be addressed before receiving cisplatin‐based chemotherapy, such as recommending close monitoring of blood pressure and cholesterol level, and encourage smoking cessation.

In addition, clinicians should be aware that a cumulative cisplatin dose of >400 mg/m^2^ may result in disproportionally more survivors with ototoxicity than groups receiving lower doses. As noted previously, follow‐up hearing assessment guidelines exist for children given cisplatin‐based chemotherapy; however, no similar recommendations have been developed for adult‐onset cancer survivors.^10^ In Frisina et al.,^10^ the authors concluded that healthcare providers should at a minimum annually query patients about hearing status, consulting with audiologists as indicated. The increase in risk for peripheral neuropathy (1.3‐fold) when comparing 300 mg/m^2^ against 400 mg/m^2^ cumulative cisplatin dose suggests for some patients (i.e., diabetic, pianist), a physician might consider the BEPX3 regimen over the EPX4 regimen. Additional research efforts are needed to replicate and validate the genetic risk factors, and their potential for new drug development to either prevent or treat these long‐lasting neurotoxicities. Further implementation of individualized risk assessments to identify patients a priori who are more susceptible to developing neurotoxicities, thus allowing for personalized education, counseling, treatment, and monitoring plans, are needed.

#### ETHICAL APPROVAL STATEMENT

Study procedures were approved by each institution's Human Subject Review Board and conducted in accordance with the U.S. Common Rule. All participants provided written consent for study participation, access to medical records, and genotyping.

## Supporting information


DataS1

## Data Availability

In addition to GWAS data in Supplemental Table 3, 5 and 6, full summary statistics for GWAS of cisplatin‐induced hearing loss, GWAS of cisplatin‐induced tinnitus and GWAS of cisplatin‐induced peripheral neuropathy are available (https://apps.cancer.iu.edu/platinum/published‐research.php).
