# Supplementary material for: Pharmacogenomics of cisplatin‐induced neurotoxicities: Hearing loss, tinnitus, and peripheral sensory neuropathy
Source: Cancer Med. 2022 Mar 23;11(14):2801–16. doi: 10.1002/cam4.4644 (PMC9302309; doi:10.1002/cam4.4644)
Supplement: Supplementary file 1 — DataS1 [file CAM4-11-2801-s001.docx]

**Supplemental Material for**

**Pharmacogenomics of Cisplatin-Induced Neurotoxicities: Hearing Loss, Tinnitus and Peripheral Sensory Neuropathy**

Xindi Zhang^1^, Matthew R. Trendowski^1^, Emma Wilkinson^1^, Mohammad Shahbazi^1^, Paul Dinh^2^, Megan M. Shuey^3^, Regeneron Genetics Center^4^, Darren R. Feldman^5^, Robert J. Hamilton^6^, David J. Vaughn^7^, Chunkit Fung^8^, Christian Kollmannsberger^9^, Robert Huddart^10^, Neil E. Martin^11^, Victoria A. Sanchez^12^, Robert D. Frisina^13^, Lawrence H. Einhorn^2^, Nancy J. Cox^3^, Lois B. Travis^2^, M. Eileen Dolan^1^

^1^Department of Medicine, University of Chicago, Chicago, IL, USA; ^2^Department of Medical Oncology, Indiana University, Indianapolis, IN; ^3^Department of Medicine, Vanderbilt University Medical Center, Nashville, TN;

^4^ Regeneron Genetics Center (RGC) authors/contributors are listed in alphabetical order.

**RGC Management and Leadership Team:** Goncalo Abecasis, Ph.D., Aris Baras, M.D., Michael Cantor, M.D., Giovanni Coppola, M.D., Aris Economides, Ph.D., Luca A. Lotta, M.D., Ph.D., John D. Overton, Ph.D., Jeffrey G. Reid, Ph.D., Alan Shuldiner, M.D.

Contribution: All authors contributed to securing funding, study design and oversight. All authors reviewed the final version of the manuscript.

**Sequencing and Lab Operations**: Christina Beechert, Caitlin Forsythe, M.S., Erin D. Fuller, Zhenhua Gu, M.S., Michael Lattari, Alexander Lopez, M.S., John D. Overton, Ph.D., Thomas D. Schleicher, M.S., Maria Sotiropoulos Padilla, M.S., Louis Widom, Sarah E. Wolf, M.S., Manasi Pradhan, M.S., Kia Manoochehri, Ricardo H. Ulloa.

Contribution: C.B., C.F., A.L., and J.D.O. performed and are responsible for sample genotyping. C.B, C.F., E.D.F., M.L., M.S.P., L.W., S.E.W., A.L., and J.D.O. performed and are responsible for exome sequencing. T.D.S., Z.G., A.L., and J.D.O. conceived and are responsible for laboratory automation. M.P., K.M., R.U., and J.D.O are responsible for sample tracking and the library information management system.

**Genome Informatics:** Xiaodong Bai, Ph.D., Suganthi Balasubramanian, Ph.D., Andrew Blumenfeld, Boris Boutkov, Ph.D., Gisu Eom, Lukas Habegger, Ph.D., Alicia Hawes, B.S., Shareef Khalid, Olga Krasheninina, M.S., Rouel Lanche, Adam J. Mansfield, B.A., Evan K. Maxwell, Ph.D., Mrunali Nafde, Sean O’Keeffe, M.S., Max Orelus, Razvan Panea, Ph.D., Tommy Polanco, B.A., Ayesha Rasool, M.S., Jeffrey G. Reid, Ph.D., William Salerno, Ph.D., Jeffrey C. Staples, Ph.D.

Contribution: X.B., A.H., O.K., A.M., S.O., R.P., T.P., A.R., W.S. and J.G.R. performed and are responsible for the compute logistics, analysis and infrastructure needed to produce exome and genotype data. G.E., M.O., M.N. and J.G.R. provided compute infrastructure development and operational support. S.B., S.K., and J.G.R. provide variant and gene annotations and their functional interpretation of variants. E.M., J.S., R.L., B.B., A.B., L.H., J.G.R. conceived and are responsible for creating, developing, and deploying analysis platforms and computational methods for analyzing genomic data.

**Research Program Management:** Marcus B. Jones, Ph.D., Jason Mighty, Ph.D., Lyndon J. Mitnaul, Ph.D.

Contribution: All authors contributed to the management and coordination of all research activities, planning and execution. All authors contributed to the review process for the final version of the manuscript.

^5^Department of Medical Oncology, Memorial Sloan-Kettering Cancer Center, New York, USA, NY; ^6^Department of Surgical Oncology, Princess Margaret Cancer Centre, Toronto, ON, Canada; ^7^Department of Medicine, University of Pennsylvania, Philadelphia, PA, USA; ^8^J.P. Wilmot Cancer Institute, University of Rochester Medical Center, Rochester, NY; ^9^Division of Medical Oncology, University of British Columbia, Vancouver, BC, Canada; ^10^Royal Marsden Hospital, London, UK;  ^11^Department of Radiation Oncology, Dana-Farber Cancer Institute, Boston, MA; ^12^Department of Otolaryngology – Head and Neck Surgery, University of South Florida, Tampa, FL, USA; ^13^Departments of Medical Engineering and Communication Sciences and Disorders, Global Center for Hearing and Speech Research, University of South Florida, Tampa, FL, USA

**Supplemental Methods**

*Data Collection for Patient Characteristics from Assessments*

Using weight and height collected during a physical examination, body mass index (BMI) (kg/m2) was defined as normal, overweight, obese, or morbidly obese (<25, 25 to <30, 30 to <40, and ≥40 kg/m2, respectively). Information regarding hypertension, hypercholesterolemia, persistent dizziness/vertigo, overall health conditions, noise exposure, alcohol consumption, and tobacco use were also obtained from these questionnaires. Hypertensive TCS were those who answered “yes, current” to the question, “Have you ever taken prescription medications for high blood pressure?” TCS were regarded as having hypercholesterolemia if they answered “yes, current” to the question, “Have you ever taken prescription medications for high cholesterol?”. Survivors with persistent dizziness/vertigo were those who answered “Yes” to the question, “Persistent dizziness or vertigo?”. Self-reported health (SRH) was rated on a poor-excellent scale in response to the question, “How would you rate your health?” For association analyses, survivor responses were converted to numeric values: poor/fair=1, good=2, very good=3, excellent=4. Survivors rating their SRH fair and poor were combined due to the low frequency of each. Exposure to noise was assessed using two questions: "Have you ever had a job where you were exposed to loud noise for 5 or more hours a week? (loud noise means noise so loud that you had to speak in a raised voice to be heard)" and "Outside of a job, have you ever been exposed to steady loud noise or music for 5 or more hours a week? (examples are noise from power tools, lawn mowers, farm machinery, cars, trucks, motorcycles, or loud music)". After numeric conversion (Yes = 1, No = 0), overall noise exposure was computed as the sum of noise exposure both at work and outside of work. Survivors with both types of noise exposure were those who answered “Yes” to both questions. Excessive drinkers were defined as those who reported consuming ≥ 2 drinks/day on average in the past year to the question "During the past year, how many drinks of alcoholic beverage have you consumed on average? [1 drink = 12 oz. beer (1 can or bottle), 4 oz. glass of wine, 1 mixed drink or shot of liquor. Tobacco use was assessed as the response to the following questions: "Have you ever smoked cigarettes?" with "Yes" and "No" options; If “yes”, they were asked “how many total years have you smoked?” Chronic smokers were defined as those who reported total smoking years > 15. They were also asked "Do you currently smoke cigarettes?"

**
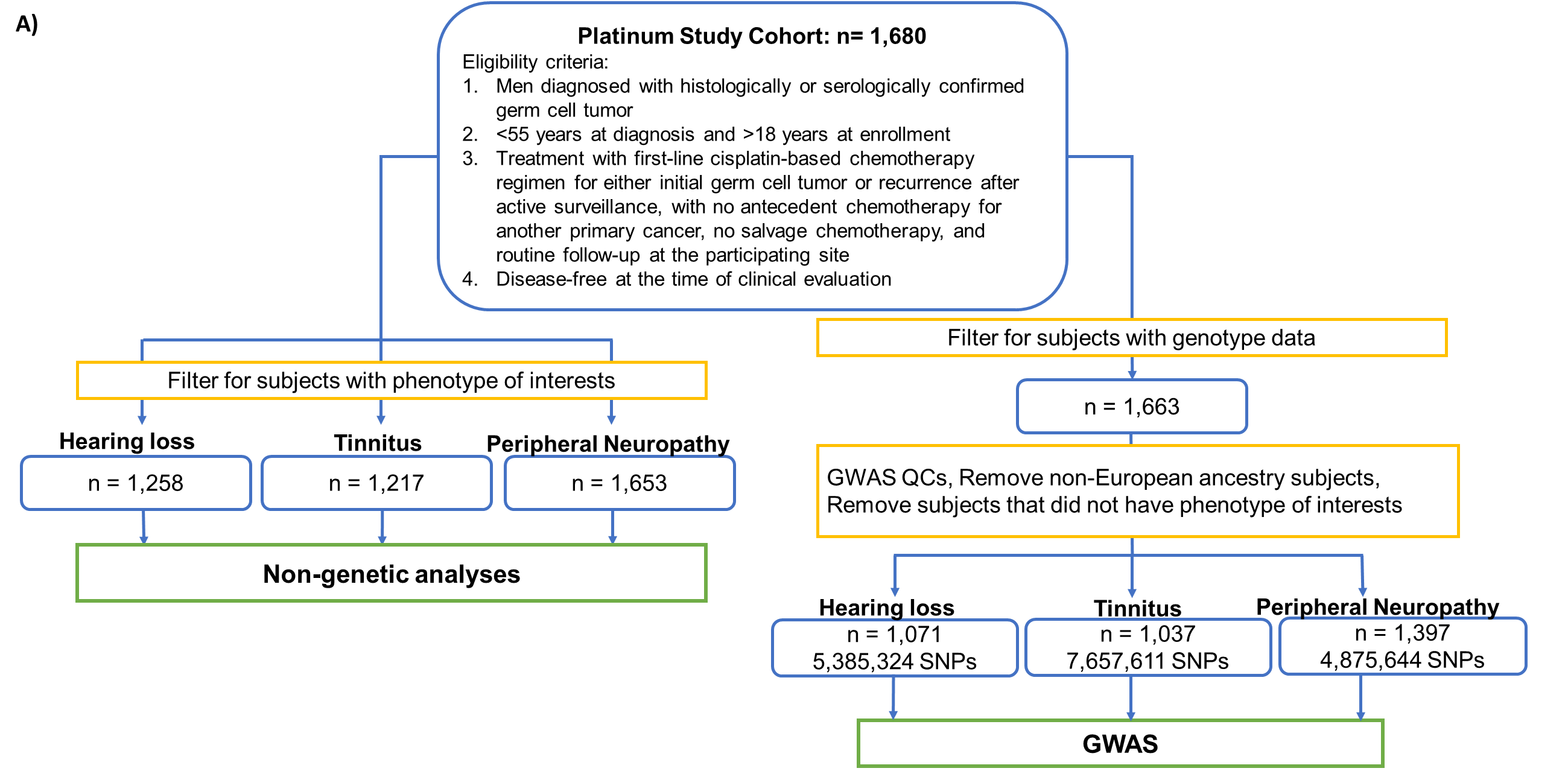
**

**
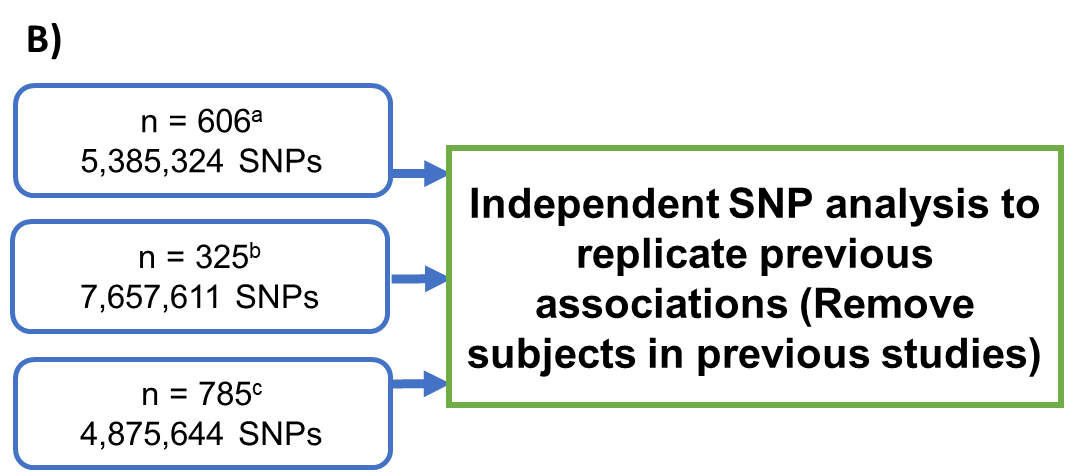
**

**Supplemental Figure 1. Overall Study Design for Studying Cisplatin-induced Neurotoxicities: Tinnitus, Hearing Loss, and Peripheral Sensory Neuropathy.** The flow chart depicts the steps and sample size of each analysis using A) the entire cohort (n=1,680) and B) the replication cohort by removing subjects in previous studies. ^a^488 subjects in the previous study(1) were excluded for hearing loss; ^b^762 subjects in the previous study(2) were excluded for tinnitus; ^c^680 subjects in the previous study(3) were excluded for peripheral sensory neuropathy.

Abbreviations: n = sample size, SNP = single nucleotide polymorphism, and GWAS = genome-wide association study.

**Supplemental Table 1. Questions to Define Peripheral Sensory Neuropathy from the EORTC-CIPN20 Questionnaire.**

| During the past 4 weeks did you have: | |
| --- | --- |
| A. | Tingling fingers or hands? |
| B. | Tingling toes or feet? |
| C. | Numbness in your fingers or hands? |
| D. | Numbness in your toes or feet? |
| E. | Shooting or burning pain in your fingers or hands? |
| F. | Shooting or burning pain in your toes or feet? |
| I. | Problems standing or walking because of  difficulty feeling the ground under your feet? |
| J. | Difficulty in distinguishing between hot and  cold water? |

**Supplemental Figure 2. Study Design for Subject and Genotype Quality Control for Cisplatin-induced Hearing Loss.** The flow chart depicts the steps used in selecting SNPs and subjects for testing in the GWAS for hearing loss. SNP = single nucleotide polymorphism, IBD = identity by descent, MAF = minor allele frequency, HWE = Hardy-Weinberg Equilibrium, and GWAS = genome-wide association study.

**
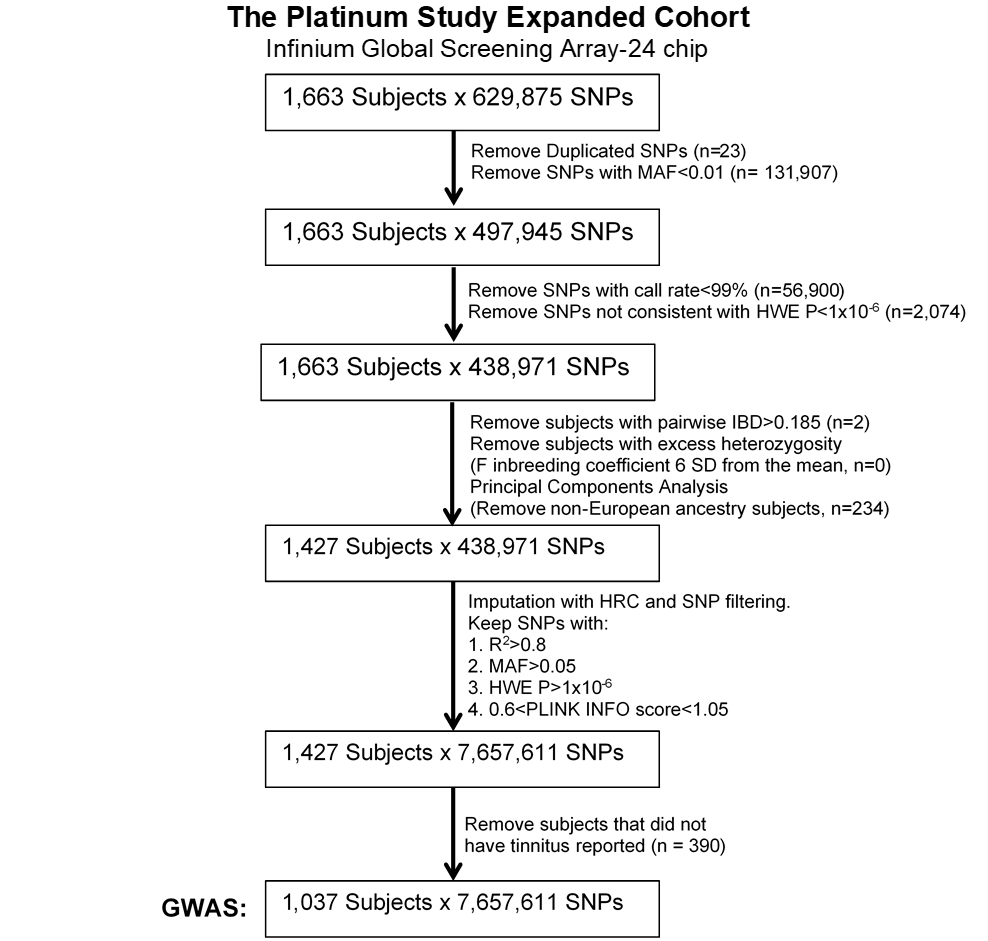
**

**Supplemental Figure 3. Study Design for Subject and Genotype Quality Control for Cisplatin-induced Tinnitus.** The flow chart depicts the steps used in selecting SNPs and subjects for testing in the GWAS for tinnitus. SNP = single nucleotide polymorphism, IBD = identity by descent, MAF = minor allele frequency, HWE = Hardy-Weinberg Equilibrium, and GWAS = genome-wide association study.

**Supplemental Figure 4. Study Design for Subject and Genotype Quality Control** **for Cisplatin-induced Peripheral Sensory Neuropathy.** The flow chart depicts the steps used in selecting SNPs and subjects for testing in the GWAS for peripheral sensory neuropathy. SNP = single nucleotide polymorphism, IBD = identity by descent, MAF = minor allele frequency, HWE = Hardy-Weinberg Equilibrium, HRC = The Haplotype Reference Consortium, and GWAS = genome-wide association study.

**Supplemental Table 2. Additional Demographic Features, Clinical Characteristics, and Patient-reported Outcomes for GWAS of 1258, 1271, and 1653 Male Germ Cell Tumor (GCT) Survivors Evaluated for Cisplatin-induced Hearing Loss, Tinnitus, and Peripheral Sensory Neuropathy**

| Characteristic | Hearing loss ^a^ | Tinnitus ^b^ | | | Peripheral sensory neuropathy ^c^ | | | |
| --- | --- | --- | --- | --- | --- | --- | --- | --- |
|  |  | All survivors | No (Controls) | Yes (Cases) | All survivors | None | Mild | Severe |
| **n** | 1,258 | 1,217 | 979 | 238 | 1,653 | 704 | 740 | 209 |
| **Categorical age at last observation, yr，** |  |  |  |  |  |  |  |  |
| <20 | 10 (0.8) | 11 (0.9) | 9 (0.9) | 2 (0.8) | 14 (0.9) | 8 (1.1) | 6 (0.8) | 0 (0.0) |
| 20-29 | 272 (21.6) | 264 (21.7) | 225 (23.0) | 39 (16.4) | 389 (23.5) | 216 (30.7) | 144 (19.4) | 29 (13.9) |
| 30-39 | 441 (35.0) | 442 (36.3) | 371 (37.9) | 71 (29.8) | 581 (35.1) | 268 (38.1) | 252 (34.0) | 61 (29.2) |
| 40-49 | 317 (25.2) | 300 (24.7) | 226 (23.1) | 74 (31.1) | 403 (24.4) | 136 (19.3) | 198 (26.8) | 69 (33.0) |
| 50-59 | 187 (14.9) | 160 (13.1) | 114 (11.6) | 46 (19.3) | 220 (13.3) | 67 (9.5) | 107 (14.5) | 46 (22.0) |
| 60-69 | 29 (2.3) | 35 (2.9) | 30 (3.1) | 5 (2.1) | 41 (2.5) | 7 (1.0) | 30 (4.1) | 4 (1.9) |
| ≥60 | 2 (0.2) | 5 (0.4) | 4 (0.4) | 1 (0.4) | 5 (0.3) | 2 (0.3) | 3 (0.4) | 0 (0.0) |
| **Categorical age at GCT diagnosis, yr** |  |  |  |  |  |  |  |  |
| <20 | 85 (6.8) | 89 (7.3) | 76 (7.8) | 13 (5.5) | 123 (7.5) | 72 (10.2) | 43 (5.8) | 8 (3.8) |
| 20-29 | 472 (37.5) | 486 (40.0) | 400 (40.0) | 86 (36.1) | 658 (39.8) | 355 (50.5) | 241 (32.6) | 62 (29.7) |
| 30-39 | 412 (32.8) | 388 (31.9) | 321 (32.8) | 67 (28.2) | 531 (32.1) | 188 (26.7) | 270 (36.5) | 73 (34.9) |
| 40-49 | 236 (18.8) | 202 (16.6) | 145 (14.8) | 57 (23.9) | 276 (16.7) | 79 (11.2) | 147 (19.9) | 50 (23.9) |
| 50-59 | 52 (4.1) | 51 (4.2) | 36 (3.7) | 15 (6.3) | 64 (3.9) | 10 (1.4) | 38 (5.2) | 16 (7.7) |
| ≥60 | 1 (0.0) | 1 (0.0) | 1 (0.0) | 0 (0.0) | 1 (0.0) | 0 (0.0) | 1 (0.0) | 0 (0.0) |
| **Categorical BMI at clinical evaluation, kg/m^2 d^** |  |  |  |  |  |  |  |  |
| Normal | 359 (28.8) | 342 (28.4) | 281 (29.0) | 61 (25.8) | 476 (29.0) | 216 (31.0) | 208 (28.3) | 52 (25.0) |
| Overweight | 519 (41.6) | 514 (42.7) | 413 (42.6) | 101 (42.8) | 685 (41.8) | 299 (42.9) | 304 (41.4) | 82 (39.4) |
| Obese | 323 (25.9) | 304 (25.2) | 240 (24.8) | 64 (27.1) | 415 (25.3) | 162 (23.2) | 193 (26.3) | 60 (28.8) |
| Morbidly obese | 47 (3.8) | 45 (3.7) | 35 (3.6) | 10 (4.2) | 63 (3.8) | 20 (2.9) | 29 (4.0) | 14 (6.7) |
| **Number of cycles of platinum-based chemotherapy ^e^** |  |  |  |  |  |  |  |  |
| <= 2 | 35 (2.7) | 47 (3.9) | 42 (4.3) | 5 (2.1) | 58 (3.5) | 27 (3.9) | 29 (4.0) | 2 (1.0) |
| 3 | 506 (40.3) | 484 (39.9) | 402 (41.2) | 82 (34.6) | 650 (39.4) | 300 (42.7) | 295 (39.9) | 55 (26.4) |
| 4 | 686 (54.6) | 639 (52.7) | 509 (52.2) | 130 (54.9) | 884 (53.6) | 352 (50.1) | 394 (53.3) | 138 (66.3) |
| >4 | 30 (2.4) | 43 (3.5) | 23 (2.3) | 20 (8.4) | 57 (3.5) | 23 (3.3) | 21 (2.8) | 13 (6.3) |
| **Tinnitus ^bf^** |  |  |  |  |  |  |  |  |
| Yes | 180 (19.8) | -- | -- | -- | 238 (19.6) | 55 (9.7) | 120 (23.5) | 63 (45.3) |
| No | 728 (80.2) | -- | -- | -- | 979 (80.4) | 512 (90.3) | 391 (76.5) | 76 (54.7) |
| **Peripheral sensory neuropathy ^cg^** |  |  |  |  |  |  |  |  |
| None | 527 (42.3) | 567 (46.6) | 512 (52.3) | 55 (23.1) | -- | -- | -- | -- |
| Mild | 555 (44.6) | 511 (42.0) | 391 (39.9) | 120 (50.4) | -- | -- | -- | -- |
| Severe | 163 (13.1) | 139 (11.4) | 76 (7.8) | 63 (26.5) | -- | -- | -- | -- |
| **Audiometrically assessed hearing loss ^h^** |  |  |  |  |  |  |  |  |
| Normal | -- | 503 (55.4) | 462 (63.5) | 41 (22.8) | 674 (54.1) | 339 (64.3) | 278 (50.1) | 57 (35.0) |
| Mild | -- | 237 (26.1) | 185 (25.4) | 52 (28.9) | 333 (26.8) | 122 (23.1) | 153 (27.6) | 58 (35.6) |
| Moderate | -- | 95 (10.5) | 55 (7.5) | 40 (22.2) | 132 (10.6) | 41 (7.8) | 66 (11.9) | 25 (15.3) |
| Moderately Severe | -- | 53 (5.8) | 19 (2.6) | 34 (18.9) | 77 (6.2) | 22 (4.2) | 39 (7.0) | 16 (9.8) |
| Severe | -- | 16 (1.8) | 5 (0.7) | 11 (6.1) | 24 (1.9) | 2 (0.4) | 16 (2.9) | 6 (3.7) |
| Profound | -- | 4 (0.4) | 2 (0.3) | 2 (1.1) | 5 (0.4) | 1 (0.2) | 3 (0.5) | 1 (0.6) |
| **Race ^i^** |  |  |  |  |  |  |  |  |
| White | 1,054 (86.3) | 1038 (87.4) | 823 (86.4) | 215 (91.5) | 1,396 (86.5) | 592 (86.8) | 629 (86.3) | 175 (86.6) |
| Other | 168 (13.7) | 149 (12.6) | 129 (13.6) | 20 (8.5) | 217 (13.5) | 90 (13.2) | 100 (13.7) | 27 (13.4) |
| **Overall noise exposure** |  |  |  |  |  |  |  |  |
| None | 743 (59.1) | 732 (60.1) | 623 (63.6) | 109 (45.8) | 965 (58.4) | 438 (62.2) | 428 (57.8) | 99 (47.4) |
| Work-related OR other exposure | 324 (25.7) | 309 (25.4) | 236 (24.1) | 73 (30.7) | 439 (26.5) | 180 (25.6) | 200 (27.0) | 59 (28.2) |
| Work-related AND other exposure | 191 (15.2) | 176 (14.5) | 120 (12.3) | 56 (23.5) | 249 (15.1) | 86 (12.2) | 112 (15.2) | 51 (24.4) |
| **Smoking status ^j^** |  |  |  |  |  |  |  |  |
| Current smoker | 82 (6.8) | 105 (9.0) | 77 (8.2) | 28 (12.2) | 144 (9.1) | 45 (6.6) | 68 (9.6) | 31 (15.8) |
| Former smoker | 374 (31.2) | 367 (31.4) | 290 (30.8) | 77 (33.6) | 511 (32.3) | 202 (29.7) | 236 (33.3) | 73 (37.2) |
| Never smoker | 744 (62.0) | 698 (59.7) | 574 (61.0) | 124 (54.1) | 929 (58.6) | 432 (63.6) | 405 (57.1) | 92 (46.9) |
| **Chronic smoker ^k^** |  |  |  |  |  |  |  |  |
| Yes | 98 (22.4) | 111 (24.3) | 74 (20.8) | 37 (36.6) | 148 (23.3) | 37 (15.6) | 73 (24.8) | 38 (36.9) |
| No | 339 (77.6) | 345 (75.7) | 281 (79.2) | 64 (63.4) | 486 (76.7) | 200 (84.4) | 221 (75.2) | 65 (63.1) |
| **Excess drinker ^l^** |  |  |  |  |  |  |  |  |
| <2 drinks per day | 1,088 (87.8) | 1,049 (87.1) | 838 (86.6) | 211 (89.0) | 1,432 (87.4) | 626 (89.9) | 634 (86.0) | 172 (83.5) |
| >=2 drinks per day | 151 (12.2) | 156 (12.9) | 130 (13.4) | 26 (11.0) | 207 (12.6) | 70 (10.1) | 103 (14.0) | 34 (16.5) |

NOTE: Data presented as number (%) unless otherwise noted.

Abbreviations: BMI, body mass index

^a^ 1,258 patients were included with quantitative values modeled using the geometric mean of air conduction thresholds measured at each frequency (4, 6, 8, 10 and 12 kHz) that demonstrated a statistically significant relationship between cumulative cisplatin dose and hearing loss, after age adjustment as described previously (1,4).

^b^ Tinnitus excludes 463 participants who did not answer related questions. Cases are restricted to survivors who reported "quite a bit" or "very much" tinnitus. Survivors who reported "a little" tinnitus (n=426) are excluded from the table and all analyses.

^c^ Following conversion of the Likert scale: "none, a little, quite a bit, very much" to a 0-3 numeric scale, each individual was assigned a summary statistic for the sensory subscale [Cronbach (a=0.88) and the motor subscale (a=0.78) by taking the mean of the response in the subscale: none (mean=0), mild (0<mean<1), severe (mean>; (3)). Category/phenotype excludes 27 participants for whom the variables were not stated.

^d^ BMI was defined as the following: normal: <25 kg/m^2^; overweight: 25-30 kg/m^2^; obese: 30-40 kg/m^2^; and morbidly obese: ≥40 kg/m^2^

^e^ Number of cisplatin cycles were not state for 1 hearing loss participants, 4 tinnitus participants, and 4 peripheral sensory neuropathy participants.

^f^ Tinnitus status was not stated for 350 participants with hearing loss and 436 participants with peripheral sensory neuropathy.

^j^ Peripheral sensory neuropathy status was not stated for 13 participants with hearing loss. We created a summary statistic of peripheral neuropathy mathematically equivalent to the standard scoring algorithm (5): we took the mean of the scores, effectively summing scores and scaling to 0–3 and imputing missing data points from available ones. 34 participants were missing one response, 27 participants were missing two responses, two participants were missing 3 responses, one participant was missing four responses, and one participants was missing five responses.

^h^ ASHA criteria defined hearing loss severity as the following: mild: 21–40 dB; moderate: 41–55 dB; moderately severe: 56–70 dB; severe: 71–90 dB; and profound: >90 dB; for at least one tested frequency for either ear (<https://www.asha.org/public/hearing/Degree-of-Hearing-Loss>). We applied this definition to the geometric mean of bilateral average hearing threshold at 4, 8, 10, 12 kHz. Audiometrically assessed hearing loss was not measured for 309 participants with tinnitus and 408 participants with peripheral sensory neuropathy.

^i^ Race was not stated for 36 participants with hearing loss, 30 participants with tinnitus, and 40 participants with peripheral neuropathy; only genetically European were included in the genetic analysis (refer to Methods).

^j^ Smoking status were not reported for 58 participants with hearing loss, 47 participants with tinnitus, and 69 participants with peripheral neuropathy.

^k^ Chronic smoking category excluded participants who did not answer related question: 821 participants for hearing loss, 761 participants for tinnitus, and 1019 participants for peripheral neuropathy.

^l^ Excess drinking status were not reported for 19 participants with hearing loss, 12 participants with tinnitus, and 14 participants with peripheral neuropathy.

**Supplemental Table 3. Top 100 GWAS Results for Cisplatin-Induced Hearing Loss (P<2.83x10^-5^) in Entire Dataset.**

| CHR | BP | SNP | EFFECT | REFERENCE | β | STAT | P |
| --- | --- | --- | --- | --- | --- | --- | --- |
| 1 | 63549230 | rs1391812 | T | C | 0.1968 | 5.452 | 6.20E-08 |
| 1 | 63552112 | rs1004517 | A | G | 0.196 | 5.428 | 7.08E-08 |
| 8 | 17746374 | rs34612399 | A | G | 0.3119 | 4.891 | 1.16E-06 |
| 10 | 3138265 | rs7074733 | T | C | 0.1901 | 4.772 | 2.08E-06 |
| 2 | 227402018 | rs16825777 | T | C | 0.2816 | 4.762 | 2.19E-06 |
| 1 | 63549282 | rs953654 | T | C | -0.1604 | -4.72 | 2.68E-06 |
| 2 | 227416662 | rs11676181 | T | C | 0.27 | 4.663 | 3.51E-06 |
| 16 | 86910194 | rs11642990 | G | C | -0.218 | -4.66 | 3.57E-06 |
| 1 | 63548419 | rs10493333 | G | C | 0.1626 | 4.66 | 3.57E-06 |
| 4 | 6276630 | rs62283056 | C | G | 0.1927 | 4.599 | 4.76E-06 |
| 12 | 104638773 | rs4406890 | G | T | 0.2046 | 4.561 | 5.69E-06 |
| 5 | 134443908 | rs72802321 | C | T | -0.3614 | -4.556 | 5.81E-06 |
| 5 | 60069057 | rs13158665 | T | C | -0.1546 | -4.55 | 5.98E-06 |
| 4 | 6275735 | rs17718958 | A | G | 0.2155 | 4.538 | 6.33E-06 |
| 4 | 6276805 | rs62283057 | T | C | 0.2155 | 4.538 | 6.33E-06 |
| 12 | 104645363 | rs7960443 | A | G | 0.2042 | 4.533 | 6.46E-06 |
| 12 | 104645669 | rs6539132 | A | C | 0.2042 | 4.533 | 6.46E-06 |
| 12 | 104646770 | rs4964267 | A | G | 0.2042 | 4.533 | 6.46E-06 |
| 12 | 104649733 | rs4964728 | A | G | 0.2042 | 4.533 | 6.46E-06 |
| 12 | 104651110 | rs10861181 | C | T | 0.2042 | 4.533 | 6.46E-06 |
| 12 | 104651482 | rs7297560 | T | C | 0.2042 | 4.533 | 6.46E-06 |
| 4 | 6284965 | rs4568307 | G | A | 0.1646 | 4.498 | 7.60E-06 |
| 1 | 63550875 | rs1466834 | A | G | -0.153 | -4.492 | 7.84E-06 |
| 1 | 63550604 | rs6662149 | T | C | -0.1533 | -4.491 | 7.86E-06 |
| 4 | 6283460 | rs10028875 | T | C | 0.1647 | 4.489 | 7.93E-06 |
| 9 | 36309845 | rs72624021 | A | G | 0.1918 | 4.457 | 9.19E-06 |
| 9 | 36311610 | rs7853674 | A | G | 0.1918 | 4.457 | 9.19E-06 |
| 18 | 54490774 | rs4800952 | G | A | 0.2888 | 4.435 | 1.02E-05 |
| 4 | 6281756 | rs4328980 | G | A | 0.1628 | 4.432 | 1.03E-05 |
| 4 | 6281961 | rs752854 | T | C | 0.1628 | 4.432 | 1.03E-05 |
| 12 | 104640920 | rs10400561 | G | A | 0.2013 | 4.43 | 1.04E-05 |
| 12 | 104641066 | rs10745994 | G | A | 0.2013 | 4.43 | 1.04E-05 |
| 12 | 104641092 | rs7958851 | C | T | 0.2013 | 4.43 | 1.04E-05 |
| 1 | 63549797 | rs12749598 | T | C | -0.1515 | -4.427 | 1.06E-05 |
| 10 | 125956348 | rs11245310 | A | G | 0.1767 | 4.425 | 1.07E-05 |
| 12 | 104650735 | rs11111955 | C | T | 0.2018 | 4.423 | 1.08E-05 |
| 12 | 104652398 | rs4964270 | G | A | 0.2018 | 4.423 | 1.08E-05 |
| 12 | 104652441 | rs4964271 | C | T | 0.2018 | 4.423 | 1.08E-05 |
| 11 | 12538367 | rs7932845 | G | T | -0.2587 | -4.422 | 1.08E-05 |
| 4 | 6282393 | rs1079215 | G | A | 0.1614 | 4.416 | 1.11E-05 |
| 14 | 48383245 | rs7154446 | C | T | -0.2898 | -4.409 | 1.15E-05 |
| 14 | 48384025 | rs715994 | G | C | -0.2898 | -4.409 | 1.15E-05 |
| 14 | 48387621 | rs9672030 | A | G | -0.2898 | -4.409 | 1.15E-05 |
| 18 | 3461224 | rs11873061 | C | T | -0.1999 | -4.402 | 1.18E-05 |
| 4 | 6302519 | rs1801212 | A | G | 0.1703 | 4.395 | 1.22E-05 |
| 4 | 6283253 | rs9997824 | C | T | 0.1608 | 4.39 | 1.25E-05 |
| 4 | 6284633 | rs10001190 | G | A | 0.1593 | 4.382 | 1.29E-05 |
| 12 | 104645405 | rs7975161 | C | T | 0.1999 | 4.377 | 1.32E-05 |
| 6 | 12088677 | rs9462452 | C | A | -0.1546 | -4.358 | 1.44E-05 |
| 22 | 46403715 | rs28698504 | A | G | 0.1682 | 4.35 | 1.49E-05 |
| 4 | 6287456 | rs4688988 | T | C | 0.172 | 4.345 | 1.53E-05 |
| 4 | 6286087 | rs4234729 | C | T | 0.1718 | 4.344 | 1.53E-05 |
| 4 | 6304286 | rs1046319 | T | C | 0.1669 | 4.325 | 1.67E-05 |
| 18 | 3460915 | rs12954964 | G | T | -0.1962 | -4.324 | 1.68E-05 |
| 16 | 65881254 | rs27337 | A | G | 0.2398 | 4.318 | 1.72E-05 |
| 22 | 45125757 | rs3788611 | A | G | -0.2072 | -4.317 | 1.73E-05 |
| 13 | 67755970 | rs55982438 | T | A | 0.2158 | 4.309 | 1.79E-05 |
| 14 | 84694899 | rs1462338 | C | T | -0.2032 | -4.302 | 1.85E-05 |
| 12 | 52007460 | rs77430639 | A | G | -0.244 | -4.3 | 1.86E-05 |
| 12 | 52024169 | rs7296719 | A | G | -0.244 | -4.3 | 1.86E-05 |
| 1 | 63553899 | rs6661226 | C | G | -0.1468 | -4.298 | 1.88E-05 |
| 16 | 86896940 | rs12926612 | A | C | -0.1846 | -4.298 | 1.89E-05 |
| 10 | 23408278 | rs7427 | T | C | -0.1543 | -4.297 | 1.89E-05 |
| 9 | 2694050 | rs72692592 | C | G | -0.2243 | -4.295 | 1.91E-05 |
| 9 | 2694825 | rs72692594 | T | C | -0.2243 | -4.295 | 1.91E-05 |
| 11 | 34273669 | rs10836169 | T | G | -0.1859 | -4.292 | 1.94E-05 |
| 9 | 2694290 | rs748784 | C | G | -0.2237 | -4.287 | 1.97E-05 |
| 9 | 2694347 | rs748785 | C | T | -0.2237 | -4.287 | 1.97E-05 |
| 9 | 2694436 | rs748786 | T | C | -0.2237 | -4.287 | 1.97E-05 |
| 9 | 2694468 | rs2009124 | A | G | -0.2237 | -4.287 | 1.97E-05 |
| 14 | 53160744 | rs12888246 | T | A | -0.1468 | -4.279 | 2.05E-05 |
| 14 | 84648309 | rs67385182 | G | C | -0.2022 | -4.278 | 2.06E-05 |
| 22 | 45124846 | rs3761481 | T | C | -0.2034 | -4.274 | 2.09E-05 |
| 14 | 84687137 | rs66781263 | A | G | -0.2021 | -4.273 | 2.10E-05 |
| 14 | 84689312 | rs34810076 | G | A | -0.2021 | -4.273 | 2.10E-05 |
| 4 | 6285715 | rs4688985 | G | A | 0.169 | 4.272 | 2.11E-05 |
| 4 | 6295985 | rs4689395 | A | G | 0.1568 | 4.27 | 2.13E-05 |
| 2 | 227414795 | rs11684196 | G | A | 0.2992 | 4.255 | 2.28E-05 |
| 12 | 18799560 | rs522034 | C | T | -0.1628 | -4.255 | 2.28E-05 |
| 14 | 84695983 | rs34390169 | T | C | -0.2009 | -4.251 | 2.32E-05 |
| 14 | 84642258 | rs71418949 | T | C | -0.201 | -4.249 | 2.33E-05 |
| 14 | 84648164 | rs12887555 | G | A | -0.201 | -4.249 | 2.33E-05 |
| 4 | 7822394 | rs78512616 | A | G | -0.3307 | -4.241 | 2.42E-05 |
| 20 | 34811044 | rs2104010 | T | C | 0.1575 | 4.232 | 2.51E-05 |
| 8 | 124335015 | rs76098909 | C | T | -0.2295 | -4.231 | 2.53E-05 |
| 4 | 6314784 | rs4333261 | T | C | 0.1598 | 4.23 | 2.54E-05 |
| 14 | 84639111 | rs36046793 | T | A | -0.1989 | -4.226 | 2.59E-05 |
| 4 | 6295064 | rs10755148 | G | A | 0.1539 | 4.224 | 2.61E-05 |
| 12 | 97806276 | rs1579196 | T | C | -0.1842 | -4.224 | 2.61E-05 |
| 10 | 56482890 | rs72794555 | A | G | -0.2128 | -4.222 | 2.63E-05 |
| 16 | 86921530 | rs11641193 | T | C | -0.1873 | -4.221 | 2.64E-05 |
| 1 | 33658887 | rs2935997 | C | G | -0.3346 | -4.218 | 2.68E-05 |
| 9 | 2695130 | rs113893919 | T | C | -0.2295 | -4.217 | 2.69E-05 |
| 12 | 18803034 | rs2931484 | T | C | -0.1592 | -4.215 | 2.71E-05 |
| 5 | 42666104 | rs4419597 | C | T | 0.1824 | 4.215 | 2.72E-05 |
| 4 | 6281458 | rs11727100 | C | A | 0.1669 | 4.212 | 2.75E-05 |
| 4 | 6281496 | rs4476672 | G | A | 0.1669 | 4.212 | 2.75E-05 |
| 16 | 86879475 | rs12444737 | T | C | -0.2366 | -4.212 | 2.75E-05 |
| 2 | 212586244 | rs4673629 | T | C | 0.1469 | 4.206 | 2.83E-05 |

| **Drug** | Spearman’s rank-order correlation | | Linear regression | |
| --- | --- | --- | --- | --- |
|  | ρ | P | R^2^ | P |
| 5-FU | 0.2 | 0.1 | 0.07 | 0.08 |
| Bleomycin | 0.009 | 1.0 | 0.0007 | 0.9 |
| Bortezomib | 0.1 | 0.6 | 0.04 | 0.4 |
| Cisplatin | 0.4 | **0.04** | 0.1 | **0.03** |
| Cytarabine | 0.1 | 0.4 | 0.01 | 0.5 |
| Docetaxel | -0.04 | 0.8 | 0.0002 | 0.9 |
| Etoposide | 0.2 | 0.2 | 0.05 | 0.1 |
| Vinblastine | 0.04 | 0.8 | 0.008 | 0.6 |

**Supplemental Table 4. Correlations Between *TXNRD1* Expression and Anticancer Drug Area under the Curve (AUC) Values in CNS Tumor Cell Lines.**

Significance values were calculated using the Spearman’s rank-order correlation and linear regression. P-values<0.05 are highlighted in bold.

**Supplemental Table 5. Top 100 GWAS Results for Cisplatin-Induced Tinnitus (P<1.60x10^-5^) of Entire Dataset.**

| CHR | BP | SNP | EFFECT | REFERENCE | OR | STAT | P |
| --- | --- | --- | --- | --- | --- | --- | --- |
| 10 | 122238850 | rs4752423 | A | G | 0.4202 | -5.109 | 3.24E-07 |
| 10 | 122238794 | rs4752422 | A | C | 0.4223 | -5.082 | 3.74E-07 |
| 10 | 122239793 | rs1475373 | G | C | 0.4223 | -5.082 | 3.74E-07 |
| 10 | 122239999 | rs4237520 | C | T | 0.4223 | -5.082 | 3.74E-07 |
| 7 | 14200667 | rs11983244 | A | C | 2.97 | 5.041 | 4.62E-07 |
| 1 | 234826975 | rs607708 | A | T | 1.753 | 4.759 | 1.95E-06 |
| 1 | 234827009 | rs512578 | C | A | 1.753 | 4.759 | 1.95E-06 |
| 1 | 234827824 | rs541021 | A | T | 1.746 | 4.731 | 2.23E-06 |
| 21 | 38901620 | rs13051500 | A | C | 1.803 | 4.719 | 2.37E-06 |
| 21 | 38902379 | rs13052357 | A | C | 1.797 | 4.701 | 2.58E-06 |
| 20 | 4711755 | rs117098605 | T | G | 4.462 | 4.692 | 2.71E-06 |
| 4 | 162489247 | rs76102346 | T | C | 7.686 | 4.64 | 3.48E-06 |
| 1 | 234829747 | rs655506 | G | T | 1.72 | 4.627 | 3.72E-06 |
| 1 | 234830361 | rs2770371 | C | T | 1.72 | 4.627 | 3.72E-06 |
| 1 | 234830590 | rs498518 | A | G | 1.718 | 4.612 | 3.99E-06 |
| 3 | 33131888 | rs112303980 | A | G | 2.657 | 4.591 | 4.41E-06 |
| 19 | 10020385 | rs75764500 | T | C | 2.579 | 4.587 | 4.49E-06 |
| 13 | 24515624 | rs2765107 | A | G | 1.788 | 4.565 | 5.01E-06 |
| 1 | 234820331 | rs490559 | A | G | 0.5829 | -4.529 | 5.91E-06 |
| 1 | 234820419 | rs491427 | C | T | 0.5829 | -4.529 | 5.91E-06 |
| 1 | 234820827 | rs588965 | T | G | 0.5829 | -4.529 | 5.91E-06 |
| 9 | 31848524 | rs75341355 | A | G | 2.518 | 4.521 | 6.15E-06 |
| 13 | 24514328 | rs2810669 | T | C | 1.778 | 4.52 | 6.19E-06 |
| 13 | 24514518 | rs2765105 | T | G | 1.778 | 4.52 | 6.19E-06 |
| 13 | 24515162 | rs2765106 | C | A | 1.778 | 4.52 | 6.19E-06 |
| 7 | 22097807 | rs139884628 | C | T | 4.066 | 4.505 | 6.65E-06 |
| 13 | 65270250 | rs78597370 | A | G | 2.898 | 4.501 | 6.77E-06 |
| 13 | 65275989 | rs141170839 | A | G | 2.898 | 4.501 | 6.77E-06 |
| 4 | 99557766 | rs846004 | A | T | 5.919 | 4.493 | 7.04E-06 |
| 14 | 37480996 | rs751202 | T | A | 1.818 | 4.47 | 7.82E-06 |
| 14 | 37481442 | rs909015 | G | C | 1.818 | 4.47 | 7.82E-06 |
| 14 | 37481812 | rs17105951 | A | G | 1.818 | 4.47 | 7.82E-06 |
| 3 | 33064935 | rs7610270 | G | C | 2.462 | 4.467 | 7.94E-06 |
| 16 | 13124884 | rs10459770 | G | C | 4.236 | 4.459 | 8.22E-06 |
| 16 | 13125741 | rs7203886 | G | A | 4.236 | 4.459 | 8.22E-06 |
| 16 | 13126708 | rs76477961 | G | C | 4.236 | 4.459 | 8.22E-06 |
| 16 | 13127010 | rs7190439 | A | G | 4.236 | 4.459 | 8.22E-06 |
| 3 | 33079760 | rs78672100 | C | A | 2.457 | 4.459 | 8.24E-06 |
| 2 | 8549456 | rs4425054 | C | T | 0.3083 | -4.448 | 8.65E-06 |
| 19 | 9990729 | rs150211563 | G | A | 2.625 | 4.445 | 8.81E-06 |
| 16 | 13133980 | rs79792779 | C | G | 3.376 | 4.44 | 9.01E-06 |
| 14 | 37459015 | rs2022730 | C | T | 1.792 | 4.433 | 9.28E-06 |
| 21 | 38902907 | rs35682991 | T | C | 1.744 | 4.432 | 9.34E-06 |
| 21 | 38722674 | rs2835700 | G | T | 1.728 | 4.425 | 9.63E-06 |
| 21 | 38892198 | rs8131077 | G | C | 1.734 | 4.418 | 9.97E-06 |
| 14 | 37474637 | rs925999 | T | C | 1.801 | 4.416 | 1.01E-05 |
| 16 | 13131741 | rs7184906 | T | C | 4.283 | 4.412 | 1.03E-05 |
| 14 | 37470214 | rs61989484 | A | G | 1.799 | 4.405 | 1.06E-05 |
| 13 | 24502288 | rs1325673 | T | C | 1.838 | 4.398 | 1.09E-05 |
| 13 | 24503312 | rs17341039 | C | T | 1.838 | 4.398 | 1.09E-05 |
| 5 | 137360166 | rs62383467 | A | T | 1.675 | 4.391 | 1.13E-05 |
| 5 | 137422329 | rs217268 | A | C | 1.674 | 4.387 | 1.15E-05 |
| 1 | 234819885 | rs486650 | T | C | 1.649 | 4.376 | 1.21E-05 |
| 8 | 87163484 | rs201825788 | A | C | 3.555 | 4.373 | 1.23E-05 |
| 5 | 137401737 | rs13179129 | A | T | 1.672 | 4.373 | 1.23E-05 |
| 3 | 122503168 | rs62261679 | A | G | 4.543 | 4.372 | 1.23E-05 |
| 10 | 22859811 | rs141382055 | A | C | 3.194 | 4.364 | 1.28E-05 |
| 13 | 24513704 | rs1969882 | G | T | 1.742 | 4.363 | 1.28E-05 |
| 5 | 137371458 | rs10515497 | T | C | 1.671 | 4.362 | 1.29E-05 |
| 5 | 137381550 | rs11242413 | T | C | 1.671 | 4.362 | 1.29E-05 |
| 5 | 137426447 | rs6596422 | A | G | 1.67 | 4.36 | 1.30E-05 |
| 14 | 37480102 | rs28540719 | A | G | 1.785 | 4.352 | 1.35E-05 |
| 4 | 162487042 | rs78176588 | C | T | 6.849 | 4.349 | 1.37E-05 |
| 14 | 37471718 | rs11848465 | T | C | 1.789 | 4.349 | 1.37E-05 |
| 18 | 8627637 | rs12458216 | C | T | 2.172 | 4.346 | 1.39E-05 |
| 5 | 137401936 | rs35115838 | G | A | 1.666 | 4.343 | 1.40E-05 |
| 5 | 137402811 | rs13184628 | C | T | 1.666 | 4.343 | 1.40E-05 |
| 5 | 137403944 | rs4835758 | G | A | 1.666 | 4.343 | 1.40E-05 |
| 5 | 137404144 | rs4835759 | G | A | 1.666 | 4.343 | 1.40E-05 |
| 5 | 137405259 | rs12659908 | A | G | 1.666 | 4.343 | 1.40E-05 |
| 5 | 137409182 | rs62383476 | T | C | 1.666 | 4.343 | 1.40E-05 |
| 5 | 137409755 | rs56353546 | A | G | 1.666 | 4.343 | 1.40E-05 |
| 5 | 137411817 | rs11746900 | C | T | 1.666 | 4.343 | 1.40E-05 |
| 5 | 137412684 | rs11242414 | G | A | 1.666 | 4.343 | 1.40E-05 |
| 5 | 137412786 | rs11242415 | A | G | 1.666 | 4.343 | 1.40E-05 |
| 5 | 137413625 | rs12656356 | A | C | 1.666 | 4.343 | 1.40E-05 |
| 5 | 137416551 | rs12188416 | G | T | 1.666 | 4.343 | 1.40E-05 |
| 5 | 137417869 | rs4835761 | A | G | 1.666 | 4.343 | 1.40E-05 |
| 5 | 137427274 | rs3860103 | C | G | 1.666 | 4.343 | 1.40E-05 |
| 5 | 137428087 | rs2306110 | G | T | 1.666 | 4.343 | 1.40E-05 |
| 5 | 137431887 | rs7728933 | C | G | 1.666 | 4.343 | 1.40E-05 |
| 5 | 137432427 | rs4835668 | G | A | 1.666 | 4.343 | 1.40E-05 |
| 5 | 137435730 | rs11741240 | G | T | 1.666 | 4.343 | 1.40E-05 |
| 6 | 158987771 | rs147411083 | A | G | 6.097 | 4.342 | 1.41E-05 |
| 5 | 137441664 | rs13166144 | A | T | 0.6003 | -4.342 | 1.41E-05 |
| 5 | 137441767 | rs4835768 | G | A | 0.6003 | -4.342 | 1.41E-05 |
| 14 | 37458034 | rs28487989 | C | T | 1.779 | 4.339 | 1.44E-05 |
| 18 | 8621317 | rs11664847 | T | A | 2.167 | 4.337 | 1.44E-05 |
| 1 | 38553721 | rs12077698 | C | G | 2.922 | 4.336 | 1.45E-05 |
| 4 | 54347716 | rs111968738 | T | C | 3.822 | 4.335 | 1.46E-05 |
| 4 | 54352988 | rs113361137 | C | T | 3.822 | 4.335 | 1.46E-05 |
| 4 | 54379087 | rs16931746 | G | A | 3.822 | 4.335 | 1.46E-05 |
| 4 | 54379087 | rs112051361 | A | C | 3.822 | 4.335 | 1.46E-05 |
| 12 | 114884380 | rs1248039 | A | G | 1.662 | 4.334 | 1.46E-05 |
| 5 | 137431501 | rs217256 | T | C | 1.662 | 4.322 | 1.54E-05 |
| 5 | 137427564 | rs217254 | C | A | 1.662 | 4.321 | 1.56E-05 |
| 5 | 137427564 | rs374275479 | ATAAC | A | 1.662 | 4.321 | 1.56E-05 |
| 3 | 33048514 | rs73828910 | C | T | 2.401 | 4.32 | 1.56E-05 |
| 3 | 33048661 | rs73826335 | T | A | 2.401 | 4.32 | 1.56E-05 |

**Supplemental Table 6. Top 100 GWAS Results for Cisplatin-Induced Peripheral Sensory Neuropathy (P<1.60x10^-5^) in Entire Dataset.**

| CHR | BP | SNP | EFFECT | REFERENCE | OR | STAT | P |
| --- | --- | --- | --- | --- | --- | --- | --- |
| 2 | 71119901 | rs7572014 | A | G | 0.548051 | -5.02773 | 4.96E-07 |
| 2 | 71115883 | rs7557020 | G | C | 0.550114 | -4.99855 | 5.78E-07 |
| 2 | 140863369 | rs112913430 | G | A | 1.954367 | 4.776238 | 1.79E-06 |
| 20 | 23085003 | rs844828 | T | C | 0.680739 | -4.77451 | 1.80E-06 |
| 20 | 23085039 | rs844829 | C | G | 0.681629 | -4.75818 | 1.95E-06 |
| 22 | 38708506 | rs56182369 | A | G | 0.441494 | -4.62886 | 3.68E-06 |
| 20 | 23103870 | rs946369 | A | G | 0.686111 | -4.62661 | 3.72E-06 |
| 4 | 57633157 | rs77008993 | A | G | 1.655163 | 4.607564 | 4.07E-06 |
| 20 | 23107438 | rs6083009 | T | C | 0.691611 | -4.6063 | 4.10E-06 |
| 20 | 23106988 | rs13045861 | A | G | 0.691965 | -4.59893 | 4.25E-06 |
| 4 | 57633290 | rs112255590 | T | C | 1.655811 | 4.589539 | 4.44E-06 |
| 22 | 38699672 | rs73154475 | G | A | 0.445512 | -4.57848 | 4.68E-06 |
| 22 | 38700948 | rs62230182 | A | G | 0.445539 | -4.57793 | 4.70E-06 |
| 22 | 38701638 | rs7293235 | T | C | 0.44547 | -4.57747 | 4.71E-06 |
| 20 | 23110036 | rs6137840 | G | A | 0.698346 | -4.57492 | 4.76E-06 |
| 4 | 57636630 | rs10032814 | C | T | 1.679403 | 4.569204 | 4.90E-06 |
| 2 | 225297612 | rs62186945 | A | C | 2.066854 | 4.56658 | 4.96E-06 |
| 20 | 23108362 | rs6137837 | A | C | 0.694371 | -4.55777 | 5.17E-06 |
| 20 | 23108152 | rs6132568 | A | G | 0.694375 | -4.55767 | 5.17E-06 |
| 2 | 71128498 | rs34327179 | T | C | 0.575347 | -4.53471 | 5.77E-06 |
| 20 | 23109202 | rs6083010 | T | C | 0.697194 | -4.50471 | 6.65E-06 |
| 13 | 109837685 | rs72658211 | A | C | 0.648754 | -4.4842 | 7.32E-06 |
| 2 | 71131391 | rs3771399 | T | G | 0.580462 | -4.46541 | 7.99E-06 |
| 2 | 71131159 | rs3771401 | C | T | 0.580526 | -4.46457 | 8.02E-06 |
| 2 | 71128229 | rs4852237 | A | G | 0.580562 | -4.46408 | 8.04E-06 |
| 2 | 71129663 | rs3755340 | A | G | 0.580584 | -4.46381 | 8.05E-06 |
| 4 | 57632451 | rs78849116 | A | G | 1.612291 | 4.461432 | 8.14E-06 |
| 20 | 23110911 | rs6083012 | A | G | 0.692868 | -4.43728 | 9.11E-06 |
| 13 | 109834179 | rs55837916 | A | T | 0.652488 | -4.43234 | 9.32E-06 |
| 2 | 71127696 | rs3732243 | T | G | 0.583079 | -4.43179 | 9.35E-06 |
| 2 | 71125441 | rs3755342 | A | G | 0.583799 | -4.42175 | 9.79E-06 |
| 14 | 52132331 | rs6572783 | G | A | 0.719956 | -4.40303 | 1.07E-05 |
| 1 | 83162219 | rs392275 | T | C | 0.705383 | -4.38387 | 1.17E-05 |
| 7 | 566951 | rs144930799 | A | G | 0.59954 | -4.37672 | 1.20E-05 |
| 1 | 83138619 | rs319673 | T | C | 0.707021 | -4.37362 | 1.22E-05 |
| 1 | 83139718 | rs377406 | A | G | 0.707472 | -4.3689 | 1.25E-05 |
| 1 | 115978602 | rs533408 | G | A | 1.418436 | 4.361474 | 1.29E-05 |
| 3 | 81443513 | rs17018891 | A | G | 0.549184 | -4.35999 | 1.30E-05 |
| 1 | 83238789 | rs2389361 | T | G | 0.707131 | -4.35137 | 1.35E-05 |
| 1 | 83162083 | rs426436 | C | T | 0.71096 | -4.34985 | 1.36E-05 |
| 1 | 83139103 | rs11809889 | G | T | 0.708567 | -4.34853 | 1.37E-05 |
| 1 | 83234010 | rs7555550 | G | A | 0.70802 | -4.33908 | 1.43E-05 |
| 1 | 83233401 | rs17505362 | A | G | 0.707978 | -4.33722 | 1.44E-05 |
| 1 | 83232660 | rs12125888 | A | T | 0.708168 | -4.33655 | 1.45E-05 |
| 5 | 122637532 | rs1593922 | T | A | 0.510032 | -4.33442 | 1.46E-05 |
| 1 | 83166637 | rs2641944 | C | T | 0.716282 | -4.33268 | 1.47E-05 |
| 1 | 83147308 | rs12057639 | A | G | 0.716307 | -4.33198 | 1.48E-05 |
| 7 | 567218 | rs141305593 | G | C | 0.603291 | -4.32634 | 1.52E-05 |
| 1 | 83144898 | rs433040 | C | A | 0.712754 | -4.32504 | 1.53E-05 |
| 1 | 83145164 | rs435172 | C | T | 0.712778 | -4.32471 | 1.53E-05 |
| 1 | 83144210 | rs2641948 | G | A | 0.712779 | -4.3244 | 1.53E-05 |
| 1 | 83144740 | rs405874 | G | C | 0.712797 | -4.32439 | 1.53E-05 |
| 1 | 83144801 | rs404251 | C | G | 0.712799 | -4.32436 | 1.53E-05 |
| 1 | 83144098 | rs449899 | T | C | 0.712799 | -4.32411 | 1.53E-05 |
| 1 | 83143445 | rs450769 | T | G | 0.712799 | -4.32408 | 1.53E-05 |
| 1 | 83143521 | rs642325 | T | C | 0.712803 | -4.32403 | 1.53E-05 |
| 1 | 83143775 | rs685872 | A | G | 0.712803 | -4.32403 | 1.53E-05 |
| 1 | 83143951 | rs2787980 | A | G | 0.712805 | -4.324 | 1.53E-05 |
| 1 | 83143953 | rs2787979 | A | G | 0.712805 | -4.32399 | 1.53E-05 |
| 1 | 83141194 | rs452507 | A | C | 0.712785 | -4.32394 | 1.53E-05 |
| 1 | 83143212 | rs441237 | G | T | 0.712812 | -4.32383 | 1.53E-05 |
| 1 | 83141653 | rs436468 | A | G | 0.712809 | -4.32369 | 1.53E-05 |
| 1 | 83142847 | rs983586 | A | G | 0.712825 | -4.32358 | 1.54E-05 |
| 1 | 83141736 | rs402628 | C | T | 0.712824 | -4.32348 | 1.54E-05 |
| 1 | 83141055 | rs1655363 | C | G | 0.71284 | -4.32323 | 1.54E-05 |
| 1 | 83162337 | rs422787 | G | T | 0.712181 | -4.32134 | 1.55E-05 |
| 1 | 83167401 | rs384520 | A | G | 0.712795 | -4.31857 | 1.57E-05 |
| 1 | 83158319 | rs427959 | A | G | 0.7131 | -4.31667 | 1.58E-05 |
| 1 | 83145400 | rs380546 | G | C | 0.713448 | -4.31658 | 1.58E-05 |
| 1 | 83146165 | rs437602 | T | C | 0.713462 | -4.31643 | 1.59E-05 |
| 1 | 83145469 | rs439753 | C | T | 0.713462 | -4.31635 | 1.59E-05 |
| 1 | 83145769 | rs414558 | G | A | 0.713462 | -4.31635 | 1.59E-05 |
| 1 | 83157829 | rs376634 | C | T | 0.713203 | -4.31509 | 1.60E-05 |
| 1 | 83160435 | rs435116 | T | C | 0.713207 | -4.315 | 1.60E-05 |
| 1 | 83160594 | rs391738 | C | T | 0.713209 | -4.31497 | 1.60E-05 |
| 1 | 83160716 | rs438631 | T | C | 0.713209 | -4.31497 | 1.60E-05 |
| 1 | 83160907 | rs438845 | A | G | 0.713209 | -4.31497 | 1.60E-05 |
| 1 | 83161861 | rs407156 | T | C | 0.713209 | -4.31497 | 1.60E-05 |
| 1 | 83161931 | rs433054 | A | G | 0.713209 | -4.31497 | 1.60E-05 |
| 1 | 83160324 | rs421929 | G | T | 0.71321 | -4.31496 | 1.60E-05 |
| 1 | 83157293 | rs403657 | A | G | 0.713211 | -4.31495 | 1.60E-05 |
| 1 | 83157715 | rs394763 | C | A | 0.713211 | -4.31495 | 1.60E-05 |
| 1 | 83158517 | rs393017 | A | G | 0.713211 | -4.31495 | 1.60E-05 |
| 1 | 83158890 | rs10782792 | G | A | 0.713211 | -4.31495 | 1.60E-05 |
| 1 | 83158937 | rs2641947 | C | T | 0.713211 | -4.31495 | 1.60E-05 |
| 1 | 83159975 | rs373733 | A | G | 0.713211 | -4.31495 | 1.60E-05 |
| 1 | 83156412 | rs410028 | C | T | 0.713212 | -4.31494 | 1.60E-05 |
| 1 | 83156853 | rs434717 | T | C | 0.713212 | -4.31494 | 1.60E-05 |
| 1 | 83161076 | rs372653 | C | T | 0.713215 | -4.31484 | 1.60E-05 |
| 1 | 83161693 | rs407424 | G | A | 0.713218 | -4.31482 | 1.60E-05 |
| 1 | 83156677 | rs401816 | C | T | 0.71322 | -4.31478 | 1.60E-05 |
| 1 | 83162245 | rs427936 | A | G | 0.713259 | -4.31461 | 1.60E-05 |
| 1 | 83162609 | rs369489 | A | T | 0.713259 | -4.3146 | 1.60E-05 |
| 1 | 83164509 | rs437359 | G | T | 0.713259 | -4.3146 | 1.60E-05 |
| 1 | 83162485 | rs369855 | T | C | 0.713262 | -4.31458 | 1.60E-05 |
| 1 | 83162811 | rs433039 | C | T | 0.713262 | -4.31458 | 1.60E-05 |
| 1 | 83163316 | rs444187 | T | C | 0.713262 | -4.31458 | 1.60E-05 |
| 1 | 83163392 | rs395914 | G | A | 0.713262 | -4.31458 | 1.60E-05 |
| 1 | 83163695 | rs393614 | C | T | 0.713262 | -4.31458 | 1.60E-05 |
| 1 | 83163835 | rs384835 | T | A | 0.713262 | -4.31458 | 1.60E-05 |

**Supplemental Table 7. Replication of Previous SNPs Identified for Cisplatin-Associated Neuroxicities in Testicular Cancer Survivors.**

| **Phenotype** | **SNP** | **Ref ^*^** | **Effect** | **Reference** | **Gene** | **Discovery P-value** | **Replicated odds ratio/β** | **Replicated P-value** |
| --- | --- | --- | --- | --- | --- | --- | --- | --- |
| Cisplatin-induced hearing loss | rs62283056 | (1) | C | G | *WFS1* | 1.4x10^-8^ | 0.1 | 0.06 |
| Cisplatin-induced tinnitus | rs7606353 | (2) | G | A | *OTOS* | 1.2x10^-6^ | 0.8 | 0.7 |


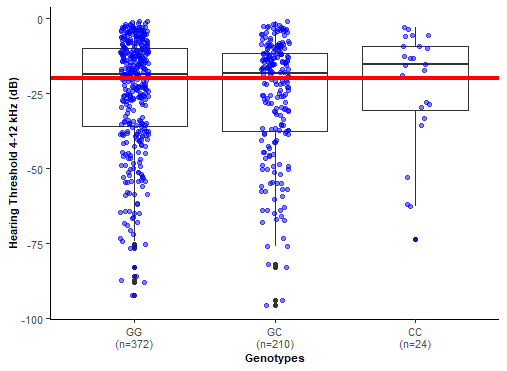


**Supplemental Figure 5. Hearing threshold distributions across rs62283056 genotypes.** Hearing loss begins at thresholds below the red line (< −20 dB). Boxes define the inter-quartile range (IQR) and the middle horizontal line represents the median. The upper whisker extends from the third quartile to the highest value that is within 1.5 × IQR. The lower whisker extends from the first quartile to the lowest value within 1.5 × IQR.

**References**

1. Wheeler HE, Gamazon ER, Frisina RD, Perez-Cervantes C, El Charif O, Mapes B*, et al.* Variants in WFS1 and Other Mendelian Deafness Genes Are Associated with Cisplatin-Associated Ototoxicity. Clin Cancer Res 2017;**23**:3325-33.

2. El Charif O, Mapes B, Trendowski MR, Wheeler HE, Wing C, Dinh PC, Jr.*, et al.* Clinical and Genome-wide Analysis of Cisplatin-induced Tinnitus Implicates Novel Ototoxic Mechanisms. Clin Cancer Res 2019;**25**:4104-16.

3. Dolan ME, El Charif O, Wheeler HE, Gamazon ER, Ardeshir-Rouhani-Fard S, Monahan P*, et al.* Clinical and Genome-Wide Analysis of Cisplatin-Induced Peripheral Neuropathy in Survivors of Adult-Onset Cancer. Clin Cancer Res 2017;**23**:5757-68.

4. Frisina RD, Wheeler HE, Fossa SD, Kerns SL, Fung C, Sesso HD*, et al.* Comprehensive Audiometric Analysis of Hearing Impairment and Tinnitus After Cisplatin-Based Chemotherapy in Survivors of Adult-Onset Cancer. J Clin Oncol 2016;**34**:2712-20.

5. Leal AD, Qin R, Atherton PJ, Haluska P, Behrens RJ, Tiber CH*, et al.* North Central Cancer Treatment Group/Alliance trial N08CA-the use of glutathione for prevention of paclitaxel/carboplatin-induced peripheral neuropathy: a phase 3 randomized, double-blind, placebo-controlled study. Cancer 2014;**120**:1890-7.
